# Shifting regional development scenarios amplify legacy phosphorus threats to water quality

**DOI:** 10.1016/j.ese.2025.100569

**Published:** 2025-05-09

**Authors:** Wei Zhan, Yedong Gao, Haoran Zhang, Yu Tian, Yanan Zou, Xiang Li, Huihang Sun, Lipin Li, Yaruo Jin, Jiaxin Cao, Yiming Liu, Nanqi Ren

**Affiliations:** a State Key Laboratory of Urban-rural Water Resource & Environment (SKLUWRE), School of Environment, Harbin Institute of Technology, Harbin, 150090, China; b China Construction Power and Environment Engineering Co., Ltd., Nanjing, 210012, China; c Heilongjiang Institute of Energy and Environment, Harbin, 150001, China

**Keywords:** Regional development pattern shift, Legacy phosphorus, Riverine pollution risk, Time-lagged response, Sustainable management strategies

## Abstract

Legacy phosphorus, accumulated from past anthropogenic activities, poses persistent and complex threats to global water quality. Despite extensive efforts to control phosphorus inputs, legacy phosphorus can persist for decades and undermine restoration goals. Emerging evidence suggests that shifts in regional development patterns profoundly reshape the dynamics and environmental risks of legacy phosphorus accumulation and mobilization. However, the mechanisms by which development pattern shifts reshape legacy phosphorus trajectories remain poorly understood. Here we show the complex pathways linking development-driven land-use changes, biogeochemical buffering capacities, and legacy phosphorus mobilization through an integrative modeling framework that couples developmental shift coefficients, anthropogenic phosphorus inventories, and riverine time-lag modeling to diagnose and predict long-term legacy phosphorus risks. Using the Songhua River as a case study, our results reveal that shifts from industrial to agricultural dominance significantly amplify legacy phosphorus accumulation by 86 times. Consequently, legacy phosphorus accounts for 65.4 %–69.9 %, surpassing current-year inputs and becoming the primary driver of riverine pollution. Furthermore, we demonstrate that development shifts systematically alter the dominant controlling factors, from fossil fuel emissions and drainage infrastructure to soil retention characteristics and agricultural practices, reshaping mitigation priorities. Our framework provides a generalizable methodology for quantifying legacy phosphorus risks under dynamic development patterns, offering immediate applications for water quality management. More broadly, this framework offers critical insights that can guide sustainable management strategies for linking evolving regional development patterns with long-term ecological restoration.

## Highlights

• A generalizable modeling framework is built to link regional development shifts with legacy phosphorus risks.
• Transitioning from industrial to agricultural dominance increases legacy phosphorus risks by 86 times.
• The legacy phosphorus levels exceed the inputs of the current year as the principal contributor to riverine pollution.
• Soil characteristics and agricultural practices dominate legacy phosphorus under agricultural intensification.
• Sustainable phosphorus management must adapt to evolving regional development patterns.

## Graphical abstract

**Figure:**
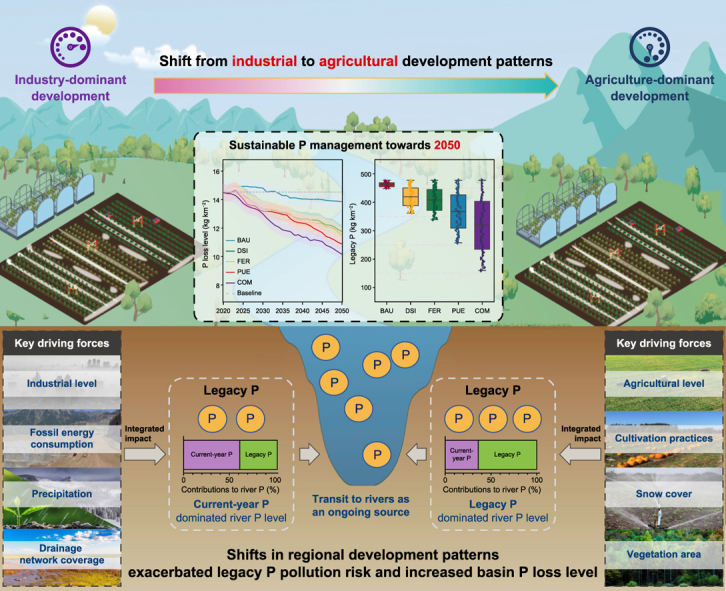

## 1 Introduction

Phosphorus (P) plays a dual role in the environment, serving as an essential nutrient for aquatic ecosystems and all terrestrial organisms and being a leading cause of eutrophication when its levels exceed natural thresholds [1,2]. P-driven eutrophication has long been identified as a critical environmental issue, particularly in both inland and coastal marine waters, where it stimulates the growth of harmful algal blooms [[3], [4], [5]]. These blooms can lead to hypoxic conditions that degrade water quality and threaten aquatic biodiversity, primarily due to the respiration of organisms and the decay of biomass produced during the bloom [6,7]. Environmental policies have been enacted globally to reduce P inputs, focusing on point sources such as wastewater treatment plants (WWTPs) to mitigate these effects. A notable success has been removing P from detergents and improving wastewater treatment, significantly reducing point-source P pollution [8,9]. However, despite these advancements, the persistent issue of non-point-source pollution and the complex dynamics of legacy P, that is, P accumulated from past human activities, continue challenging water quality restoration efforts [[10], [11], [12]].

Legacy P is defined as P retained in soils, sediments, and aquatic systems from historical anthropogenic activities [13,14]. This legacy P can persist for decades or even centuries, interacting with various biogeochemical elements such as calcium (Ca), iron (Fe), clays, and humic substances, which act as buffers that influence its mobility and potential for release [[15], [16], [17]]. These interactions determine the long-term fate of legacy P in the environment, making it a persistent and refractory pollutant that can be remobilized under specific conditions, such as heavy rainfall, soil erosion, or flash floods [7]. The remobilization of legacy P poses significant challenges to water quality, particularly as the P inputs from agricultural runoff and urban discharges continue to exacerbate nutrient loading in water bodies [[18], [19], [20]]. Therefore, even in regions where external P inputs have been reduced, legacy P continues to drive eutrophication and threatens the sustainability of freshwater systems.

In addition to natural biogeochemical processes, global climate change and increasing anthropogenic pressures have amplified the risk of legacy P immobilization in many agricultural and urbanized regions [[21], [22], [23]]. Urban areas, for example, are highly susceptible to combined sewer overflows (CSOs) during periods of intense rainfall, leading to untreated sewage entering rivers and contributing additional P loads [22]. This urban-legacy P interaction exacerbates nutrient pollution, especially in densely populated areas with insufficient stormwater management infrastructure [18,24]. Similarly, agricultural activities mobilize legacy P through soil runoff and erosion, making it crucial to understand how land-use practices interact with biogeochemical processes to influence P cycling at regional scales [25]. These dynamics underscore the need for more integrated strategies that address legacy P and ongoing inputs to effectively mitigate water quality degradation [[26], [27], [28]].

To address the complex challenges posed by legacy P, researchers have increasingly turned to modeling tools that simulate P transport, accumulation, and remobilization under different environmental and anthropogenic scenarios [14,25]. Models enable researchers to test the effectiveness of various policy interventions and land management strategies for controlling P impacts, providing valuable insights into reducing P loads and controlling eutrophication, and are essential for understanding how different factors, such as regional development patterns (i.e., agricultural and industrial development), land use, and climate change, interact to influence legacy P dynamics over time [20,24,29]. In doing so, models can offer predictive capabilities for managing P pollution under future climate and development scenarios, helping to develop more effective and sustainable management strategies for water quality improvement.

In this study, we present a detailed analysis of 40 years (1981–2020) of P dynamics in the Songhuajiang River Basin (SRB), a region that has undergone significant shifts from being one of the most industrialized regions in Northeast Asia to becoming a vital agricultural hub for China [[29], [30], [31]]. First, we tracked and visualized the regional development patterns across the SRB and evaluated both legacy and current-year anthropogenic P inputs in the same spatiotemporal dimension. Second, we quantified the response of riverine P changes to legacy inputs and identified the corresponding time lags. Third, we developed a structural model to reveal how legacy P and its key factors influence riverine P loads under development pattern shifts. Finally, we utilized machine learning to predict future scenarios for sustainable P management, offering insights into how regional and global pressures might influence legacy P risks up to 2050. This study provides a methodological framework for addressing similar legacy P risks worldwide challenges. It offers new insights into achieving a mutually beneficial outcome between changing socioeconomic development and sustainable P management.

## 2 Materials and methods

### 2.1 Study area

The SRB spans from 119°52′ to 132°31′ E and from 41°42′ to 51°38′ N and comprises a large river basin in Northeast China with an area of 557 × 10^3^ km^2^ (Supplementary Material Fig. S1) [32]. The basin includes three tributaries: the Nenjiang drainage area in the north, the Second Songhuajiang drainage area in the south, and the Mudanjiang drainage area in the southeast [31]. A total of 28 subbasins across the SRB area have been included in this study: 12 in the Nenjiang River, 4 in the Second Songhuajiang River, 2 in the Mudanjiang River, and 10 in the mainstream of the Songhuajiang River (Supplementary Materials Fig. S2–S3). More details about these subbasins are presented in Table S1 (Supplementary Material).

Located in the coldest region of China, the SRB has a temperate humid and semihumid continental monsoon climate, with monthly average temperatures fluctuating between −19 °C in January to 22 °C in July [32]. Precipitation is mainly concentrated during July and August, with an annual total of approximately 500 mm [31]. Due to the presence of fertile black soils and unique climatic conditions, the basin is suitable for large-scale cultivation of crops such as paddy rice, soybean, and corn. However, the land-use shift toward agriculture has notably expanded farmland, reducing natural landscapes (e.g., wetlands and grassland; see Supplementary Materials Fig. S2 and S15) [33]. More information on the SRB is provided in Methodology S1.1 (Supplementary Material).

### 2.2 Estimation of shifts in regional development patterns

The developmental shift coefficient was utilized to examine the shifts in regional development patterns in the SRB, whether it was industry- or agriculture-dominated. The shift was calculated by comparing the comparative concentration index of agriculture and industry [34]. Detailed data are provided in the supplementary data:

(1) $${Conindex}_{\text{Agu},i}=\frac{{AGP}_{i}÷{GDP}_{i}}{AGP÷GDP}$$

where *Conindex*_Agu,*i*_ represents the comparative concentration index of agriculture in subbasin *i*, *AGP*_*i*_ is the agricultural gross production in subbasin *i* (100 million yuan yr^−1^), *GDP*_*i*_ denotes the gross domestic product in subbasin *i* (100 million yuan yr^−1^), *AGP* is the agricultural gross production in the whole country (100 million yuan yr^−1^), and *GDP* denotes the gross domestic product in the whole country (100 million yuan yr^−1^).

(2) $${Conindex}_{\text{Indu},i}=\frac{{IGP}_{i}÷{GDP}_{i}}{IGP÷GDP}$$

where *Conindex*_Indu,*i*_ represents the comparative concentration index of industry in subbasin *i*, *IGP*_*i*_ is the industrial gross production in subbasin *i* (100 million yuan yr^−1^), *GDP*_*i*_ denotes the gross domestic product in subbasin *i* (100 million yuan yr^−1^), *IGP* is the industrial gross production in the whole country (100 million yuan yr^−1^), and *GDP* denotes the gross domestic product in the whole country (100 million yuan yr^−1^).

(3) $$Developmental\;shift\;coefficient=\frac{{Conindex}_{\text{Agu},i}}{{Conindex}_{\text{Indu},i}}$$

where *Developmental shift coefficient* is used to determine whether agriculture or industry is dominant in subbasin *i*.

### 2.3 Estimation of anthropogenic P source

The net anthropogenic P input (NAPI) was utilized to examine the anthropogenic P budget for the SRB over the period 1981–2020. This index is commonly evaluated at the national or regional level, and its large-scale calculation could lead to low uncertainty in identifying specific problems [11]. The NAPI contains five major components, namely, chemical fertilizer P application (CF), atmospheric P deposition (APD), seed P input (SI), non-food P input (NFI), and net food/feed P input (NFFI) [35]. The detailed methodologies for each P source calculation are provided in Methodologies S1.2.1–1.2.5, Fig. S4 and Table S2–S8 (Supplementary Materials).

To evaluate the uncertainty in NAPI estimation and corresponding variables, we performed Monte Carlo simulations using 10000 simulations to obtain the mean and 95 % confidence interval for the annual NAPI. We assumed that all parameters used in estimating the NAPI followed a normal distribution with a coefficient of variation of 30 % [3]. The process of estimating uncertainty was developed using Crystal Ball software, an add-on for Excel. Detailed methodologies for uncertainty assessment are provided in Methodology S1.2.6 (Supplementary Material).

### 2.4 Estimation of legacy P stock and time lags

Annual riverine P losses from the basin were calculated as the annual downstream P loads of the river (i.e., the river's export total phosphorus (TP) flux downstream). The annual riverine P load (LO_P_) was estimated as follows [36,37]:

(4) $${LO}_{P}=\sum_{i=1}^{12}\frac{Q_{i}\times C_{i}}{S}$$

where *LO*_P_ represents the annual riverine P load (kg km^−2^ yr^−1^), *Q*_*i*_ is the arithmetic average river discharge in the *i*th month (10^3^ m^3^ month^−1^), *C*_*i*_ denotes the arithmetic average TP concentration in the *i*th month (mg L^−1^), and *S* is the basin area (km^2^).

The annual legacy P was evaluated by the combination of the NAPI and LO_P_ in each year, which was calculated as follows [11,26]:

(5) $${LE}_{P,i}={NAPI}_{i}-{LO}_{p,i}$$

where ${LE}_{P,i}$ is the legacy P added in the *i*th year (kg km^−2^ yr^−1^), *NAPI*_*i*_ is the P input in the *i*th year, and *LO*_*P,i*_ is the riverine TP load in the *i*th year.

The accumulated legacy P stock (*LE*_P_) was calculated based on the accumulation of annual legacy P from 1981 to 2020 (kg km^−2^). In this study, 1981 was defined as the starting year for calculating legacy P accumulation:

(6) $${LE}_{P}=\sum_{i=1981}^{n}{NAPI}_{i}-{LO}_{p,i}$$

The cross-correlation test, a statistical method used to examine the relationships between two time-shifted variables, was employed to identify the time lags between the NAPI and riverine P loads (Methodology S1.3) [31]. The examination was performed with EViews software (Version 8).

### 2.5 Attribution analysis and future scenario prediction

To analyze the spatiotemporal variability in the contributions of P inputs to legacy P, we developed a geographical and temporal weighted regression (GTWR) model coupled with variance inflation factor analysis (Methodology S1.4; Supplementary Material Table S9). The relative contributions of the different P input sources to legacy P accumulation were further determined according to the GTWR correlation coefficients. The data processing was performed using ArcMap 10.7 and GTWR ADDIN V1.1.

Spearman's correlation analysis and the Mantel test were used to investigate relationships between legacy P and potential drivers. Before the analysis, the run-test was employed to assess the randomness of the observed variable values [31]. Based on the correlation analysis, a partial least-squares path model (PLS-SEM) was developed using SmartPLS (Version 3.3.9) to identify the key points for controlling legacy P risks and riverine P pollution (Methodology S1.6; Supplementary Material Table S13).

A new machine-learning-based model for simulating and predicting riverine P loss was developed by integrating random forest (RF) and legacy effects. Here, P input sources, climate factors, and land-use types were chosen as explanatory variables for modeling purposes. The multi-year moving average NAPI was utilized as an input for the modeling process to demonstrate the influence of legacy P on changes in riverine P loads and to enhance the model interpretability [31]. The modeling procedure was performed on the Python platform, and more details are available in Methodology S1.7 and Fig. S16 (Supplementary Material).

### 2.6 Data sources

County-level datasets, such as population, chemical fertilization rate, crop yield, livestock number, and cultivation area datasets, were adopted for estimating P inputs. These data were mainly obtained from provincial and municipal yearbooks. Other socioeconomic data (e.g., gross domestic product and sewage treatment data) were also obtained from the local Environmental Census and Agricultural Census. Riverine TP concentration and daily streamflow data were obtained from the Regional Environment Protection Agency and Hydrological Office, respectively. The climate, topography, and vegetation data were sourced from the National Earth System Science Data Center (http://www.geodata.cn/). Information on soil properties can be accessed from the Geographic Remote Sensing Ecological Network Platform (http://www.gisrs.cn/). Statistical mapping was performed with Origin 2021, ArcMap 10.7, and R Language 4.2.1.

## 3 Results and discussion

### 3.1 Shifts in regional development patterns and the impact on legacy P

**Fig. 1:**
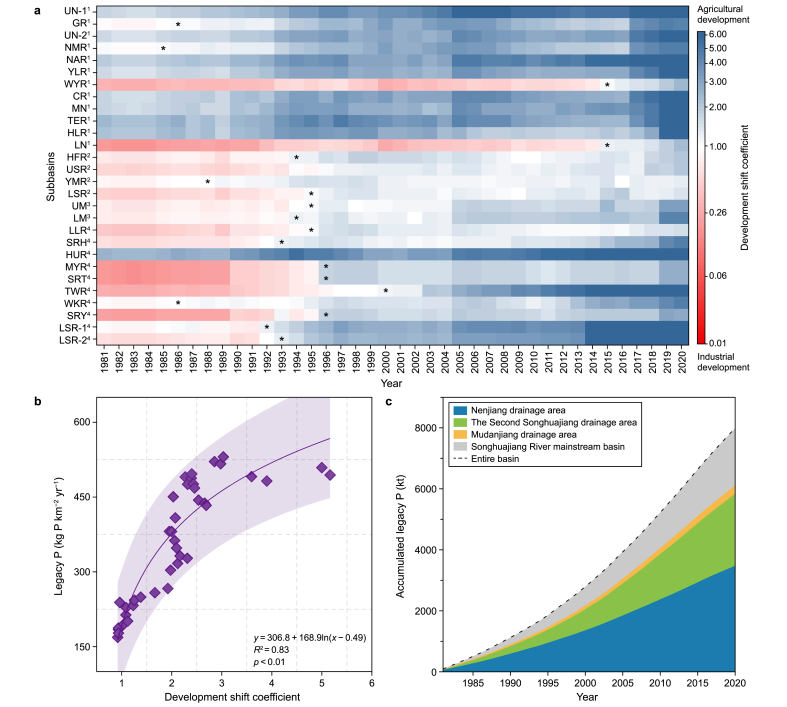
Shifts in regional development patterns and legacy P accumulation. **a**, The shift from industrial to agricultural development patterns in the Songhuajiang River Basin (SRB). Details for the subbasin acronyms can be found in Table S1 (Supplementary Materials). The superscripts of the subbasins represent the four main drainage areas of the SRB: “1” represents the Nenjiang drainage area, “2” represents the Second Songhuajiang drainage area, “3” represents the Mudanjiang drainage area, and “4” represents the Songhuajiang mainstream basin. The developmental shift coefficient reflects relative changes in the gross production between agriculture and industry, where “∗” refers to the shifts in development patterns that occurred with a coefficient of 1.0. The color variations with increasing shift coefficients in the color map are set as the “probability”. **b**, The correlation between legacy P and the developmental shift coefficient. **c**, The temporal accumulation of legacy P from 1981 to 2020. In this study, 1981 was the starting year for calculating legacy P accumulation. The “0” for accumulated legacy P before 1981 indicates the baseline for the calculations of this study.

In this study, the dynamic shifts in regional development trajectories across various subbasins within the SRB from 1981 to 2020 were explored by tracking the comparative growth rates of agricultural and industrial gross production (Fig. 1; Supplementary Material Fig. S5). The findings reveal a pronounced shift from industrial to agricultural development in the SRB, albeit with considerable spatial heterogeneity across subbasins. Specifically, most subbasins in the Nenjiang drainage area, such as Upper Nenjiang River-1 (UN-1), Upper Nenjiang River-2 (UN-2), and Namur River (NAR) had already shifted toward agricultural development before 1981. Conversely, the shift in development patterns in other areas occurred predominantly between 1992 and 1996. This shift indicates that the SRB underwent significant economic transformation during this period, strongly influenced by China's overarching development strategies.

Historically, the SRB was a focal point for advanced industrial growth in Northeast Asia before the 1980s, propelling China's socioeconomic development using resource exploitation and heavy industry [38]. The region's industrial development was primarily driven by the extraction of natural resources, including coal, oil, and metals, leading to a rapid expansion in industrial infrastructure, particularly in the energy and manufacturing sectors. However, since the 1980s, China has implemented several policy initiatives to restructure its economy. These initiatives have focused on stimulating agricultural development and transforming the SRB into a major agricultural production hub to ensure national food security and strengthen China's role in global trade. Policies such as promoting state farms, agricultural mechanization, and introducing high-yield crop varieties profoundly affected the SRB. In particular, the emphasis on sustainable agriculture and integrating modern technologies into farming practices significantly increased agricultural productivity, steering the region toward agricultural intensification as the prevailing development model. The government's shift from heavy industry towards agricultural and environmental sustainability reshaped the region's economic landscape.

Shifts in regional development patterns profoundly impact P dynamics, with legacy P accumulation in the SRB responding positively to the coefficient, indicating that the development pattern shifted (coefficient of determination *R*^2^ = 0.83, *p* < 0.01; Fig. 1b; Supplementary Material Fig. S6). Specifically, as the coefficient increased sharply (Supplementary Material Fig. S5), the accumulated legacy P in the SRB showed a near-exponential increase, increasing from 93.5 kt in 1981–7995.6 kt in 2020, an astonishing 86-fold increase (Fig. 1c). The Nenjiang drainage area, which shifted to agriculture early (Fig. 1a), accounts for the largest share of legacy P stocks (44–54 %), followed by the Second Songhuajiang drainage area (22–30 %), the Songhuajiang River mainstream basin (20–24 %), and the Mudanjiang drainage area (3.3–4.2 %; Fig. 1c; Supplementary Material Fig. S7). This distribution coincides with the time series of shifts in development patterns across subbasins in the SRB (Fig. 1a).

For the accumulated rate of legacy P, however, the Second Songhuajiang drainage area indicated the maximum value of annual legacy P, reaching 1254.7 kg km^−2^ yr^−1^ (Supplementary Material Fig. S6). This finding can be attributed to the more intensive distribution of constructed lands and farmland (Supplementary Material Fig. S2) compared to that of other areas within the SRB, exacerbating the accumulation of legacy P. Significantly, the overall accumulated intensity of legacy P in the SRB far exceeds the global average. For instance, the average annual legacy P in the SRB (356 kg km^−2^ yr^−1^ over the 1981–2020 period) was 1.2 times greater than that in the Thames River basin [39], 1.6 times greater than that in the Baltic Sea region [40], and 50 % greater than that in the Grand River Watershed [14]. This finding indicates that the SRB is a crucial area for accumulating legacy P, underscoring its vital role in global P cycles.

### 3.2 Contribution of anthropogenic P sources to legacy P accumulation

**Fig. 2:**
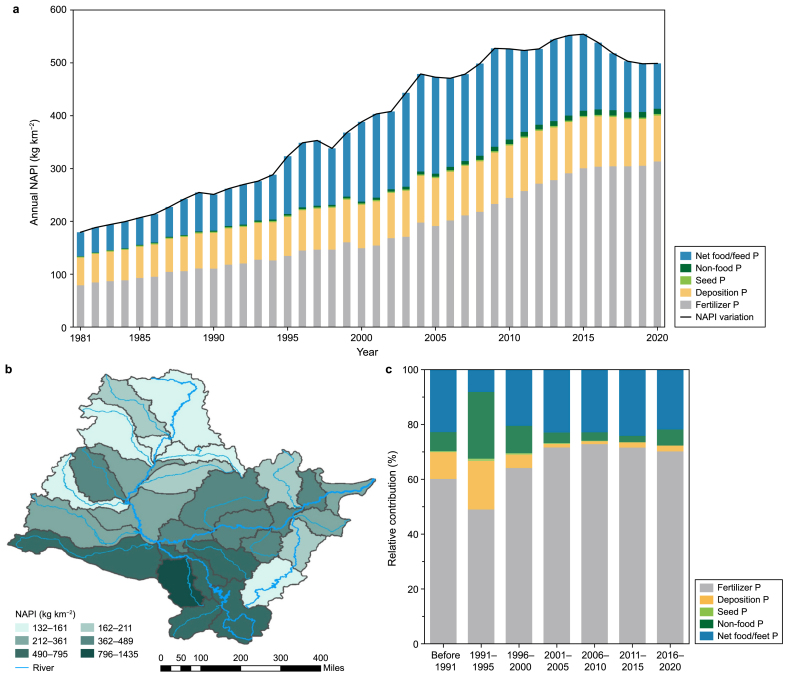
Dynamics of anthropogenic P sources and their contributions to legacy P in the context of development pattern shifts in the Songhuajiang River Basin. **a**, Historical trends of various anthropogenic P sources from 1981 to 2020. **b**, Spatial distribution of the average net anthropogenic P input (NAPI) across the basin from 1981 to 2020. **c**, Contributions of anthropogenic P sources to legacy P according to the geographical and temporal weighted regression model.

Legacy P, widely recognized as the result of P accumulation in landscapes, is driven by the disparity between NAPI and riverine outputs [11,14]. The annual NAPI in the SRB increased from 179.3 kg km^−2^ yr^−1^ in 1981 to a high of 554.2 kg km^−2^ yr^−1^ in 2015, and then decreased to 498.9 kg km^−2^ yr^−1^ by 2020 (Fig. 2a). CF emerged as the dominant contributor, with its share increasing from 44 % to 63 % over the period, highlighting the gradual intensification of agricultural practices. By contrast, the share of APD, mainly derived from industrial emissions, decreased from 29.4 % to 17.5 % as the development of the SRB shifted away from industrialization (Fig. 2a). In fact, the SRB experienced a continuous expansion of the cultivated area, increasing by 81.12 % from 2000 to 2020 alone [41].

At the same time, there was a shift toward sustainable agricultural development, with China setting a target of zero growth in chemical fertilizer usage by 2020, contributing to the decrease in NAPI of the SRB since 2015 (Fig. 2a) [11,41,42]. This underscores the continuous reshaping of the NAPI characteristics of the SRB due to changing regional development patterns, which shifted from industrial dominance to manual farming and then to intensive agriculture. In addition, spatial analysis has revealed considerable heterogeneity in NAPI across the basin, highlighting the profound influence of socioeconomic disparities on nutrient distribution in the context of similar regional development patterns [11,31]. The upper subbasins of the Nenjiang and Mudanjiang drainage areas (e.g., UN-1, UN-2, GR, NMR, and UM) exhibited NAPI levels of 132–161 kg km^−2^ yr^−1^; by contrast, the Second Songhuajiang drainage area, especially the YMR subbasin, recorded the highest NAPI, 1434.2 kg km^−2^ yr^−1^, which was 8.9–10.9 times greater than that of Nenjiang and Mudanjiang (Fig. 2b; Supplementary Materials Fig. S8 and S10).

The GTWR model was used to quantify how NAPI components influenced legacy P stocks. Fig. 2c shows that CF remained the dominant legacy P source across both the industrial and agricultural dominance periods, with its contribution increasing from 60.1 % (before 1991) to 70.2 % (2016–2020) as the basin development shifted from industrial to agricultural dominance (*p* < 0.05). This is because soils are more susceptible to P retention than paved surfaces, resulting in a greater tendency for legacy accumulation from fertilizer-derived P, even during industrial dominance [7].

By comparison, the contributions of NFI and APD to legacy P stocks were significantly influenced by regional development patterns. During the industrial dominance period, the contributions of NFI and APD increased from 6.9 % to 9.8 % (before 1991) to 24.4 % and 17.7 % (1991–1995), respectively (*p* < 0.01), while these contributions decreased to 5.8 % and 2.1 % (2016–2020), respectively, during the agricultural development period (*p* < 0.05, Fig. 2c). This phenomenon was confirmed in the subbasins of the SRB; for example, the contributions of NFI and APD decreased consistently in the Nenjiang drainage area, where most subbasins shifted to agricultural development patterns before 1981, while in the Second Songhuajiang and Mudanjiang drainage areas, their contributions increased and then decreased with regional development pattern shifts (Supplementary Material Fig. S11). Overall, our findings highlight the increasing agricultural activities and decreasing industrial footprint in shaping legacy P dynamics, suggesting a pivotal shift in environmental management priorities from controlling industrial emissions to managing agricultural P inputs to mitigating legacy pollution.

### 3.3 Temporal lags in downstream riverine P changes to legacy inputs

**Fig. 3:**
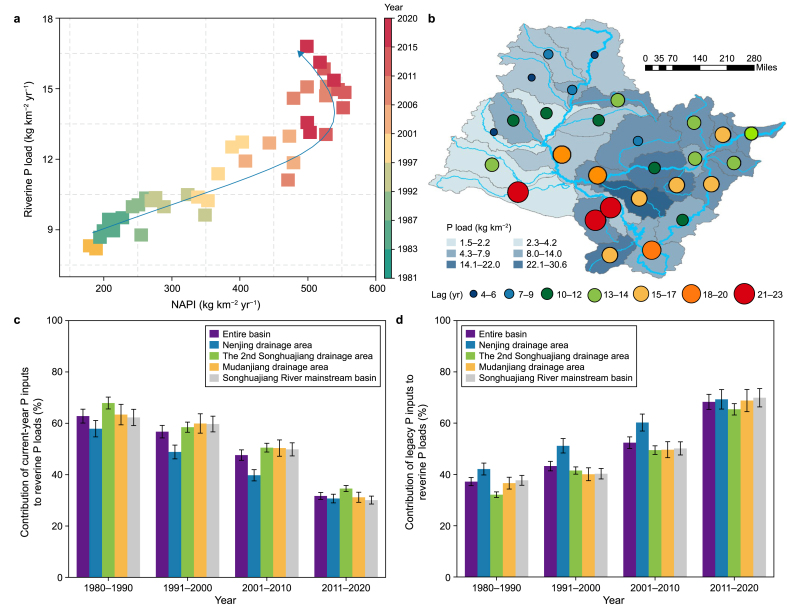
Riverine water environment response to legacy P. **a**, Time-varying relationships between the net anthropogenic P input (NAPI) and riverine P loads across the entire basin (the blue arrow is indicative, i.e., the change in arrow direction indicates that the relationship between the NAPI and riverine P loads is nonlinear over time). **b**, Geographic distributions of the riverine P load and time lag across the basin over 1981–2020 **c**, Relative contributions of the current-year P inputs to changes in the riverine P loads for the basin from 1981 to 2020. **d**, Relative contributions of legacy P inputs to changes in the riverine P loads for the basin over 1981–2020.

We investigated the lagged response of riverine water quality to legacy P by analyzing the temporal and spatial relationships between the NAPI and riverine P loads (Fig. 3a; Supplementary Material Fig. S13). Regional development patterns can lead to disparities in socioeconomic growth and human activity intensity [31], triggering spatial heterogeneity in nutrient distribution with significant impacts on riverine water quality (Fig. 2, Fig. 3b; Supplementary Material Fig. S10). Specifically, the Second Songhuajiang drainage area, marked by high NAPI due to intensive land use and agricultural activities (Fig. 2b; Supplementary Material Fig. S2 and S10), experienced greater pressure on the riverine water environment (P loads, 8–30 kg km^−2^ yr^−1^, Fig. 3b) than did the Nenjiang and Mudanjiang drainage areas (P loads, 1.5–6.1 kg km^−2^ yr^−1^). Spatial analysis from 1981 to 2020 revealed significant linear relationships between the NAPI (environmental pressure) and riverine P (riverine response) loads across the SRB, confirming that higher anthropogenic P inputs are associated with increased riverine P loads (*R*^2^ = 0.629–0.689, *p* < 0.01; Supplementary Material Fig. S12). However, the robustness of this relationship weakened over time as regional development patterns shifted toward agriculturalization, with Spearman's *ρ* decreasing from 0.689 during 1981–1990 to 0.629 during 2010–2020 (Supplementary Material Fig. S12).

This evolving dynamic is further illustrated by the changing trajectory between the NAPI and riverine P loads over time (Fig. 3a; Supplementary Material Fig. S13). From 1981 to 2010, the NAPI and riverine P loads exhibited a positive response throughout the basin, while this relationship was disrupted and shifted to a negative trend after 2010, thus forming a counterclockwise loop in the NAPI-riverine P load trajectory. This “loop” revealed a visible lag in the response of riverine P loads to NAPI, implying that riverine P loads were influenced by current-year P inputs and legacy P accumulated in previous years [25], particularly evident in the agricultural development period. Initially (i.e., 1981–1990), current-year P inputs were the main contributors to increasing riverine P loads, accounting for 62.3–67.9 % of the increase (Fig. 3c); however, as the development pattern shifted, legacy P increasingly contributed and emerged as the primary contributor, accounting for 65.4–69.9 % of the increase over 2011–2020 (Fig. 3d). This finding highlights legacy P as a long-term source affecting downstream waters, further exacerbated by the shift from industrial to agricultural dominance development.

The enduring mobilization of legacy P to rivers can introduce delays in the effectiveness of P reduction strategies [14], with lag times within the SRB ranging from 4 to 23 years and an average of 13.5 years (Fig. 3b). These lag times varied considerably between subbasins and were profoundly influenced by regional development, land use, and topography [17,26]. For instance, subbasins in the Nenjiang drainage area had shorter lag times, with CR, UN-1, and NMR having the shortest lag times of 4, 5, and 6 years (Fig. 3b), which can be attributed to the reduced legacy P intensity corresponding to low economic levels (Supplementary Materials Fig. S2 and S10), as well as steeper terrain (Supplementary Material Fig. S1) facilitating faster legacy P migration to rivers [24,43]. By contrast, central subbasins such as the YMR and LR, which experienced shifts in regional development patterns after 1981 at high economic levels, exhibited longer lag times of 23 and 21 years, respectively (Fig. 3b; Supplementary Materials Fig. S2 and S10). This disparity underscores the differential mobilization process of legacy P influenced by different regional development trajectories across the basin.

### 3.4 Complex interplay of key factors affecting legacy P dynamics

#### 3.4.1 Driving factors behind legacy P stock variations

**Fig. 4:**
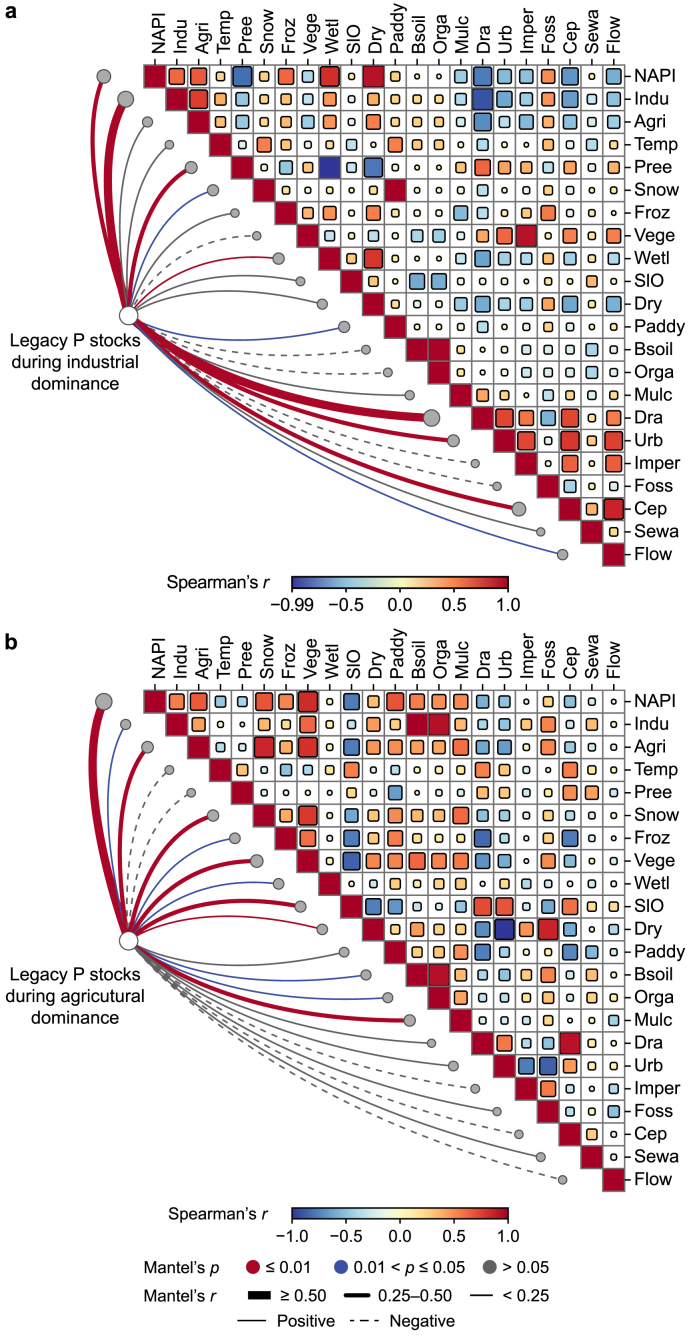
Interrelations between P cycle-related factors and legacy P dynamics before and after shifts in regional development patterns. Coefficients of Spearman's correlation and the Mantel test in the industrial development period from 1981 to 1990 (**a**) and the agricultural development period from 2010 to 2020 (**b**). Detailed information on these factors is provided in Table S10–S12 (Supplementary Materials).

To elucidate how shifts in regional development patterns influenced legacy P dynamics, we analyzed correlations between legacy P and key characteristics of the SRB across two pivotal periods: the industrial (1981–1990) and agricultural (2010–2020) development periods. As illustrated in Fig. 4 and Table S10 (Supplementary Material), consistent and significant positive correlations were observed between legacy P (Leg) and NAPI in both periods (Spearman's *ρ* = 0.686 and 0.998, respectively; *p* < 0.01). In addition, both industrial (Indu) and agricultural (Agri) levels demonstrated strong positive correlations with legacy P during each timeframe (Spearman's *ρ* > 0.5; *p* < 0.01). These relationships underscore the direct link between increased P inputs from industrial and agricultural growth and the escalation of legacy P stocks [44].

During the industrial dominance period, legacy P was positively correlated with fossil energy consumption (Foss) (Spearman's *ρ* = 0.536; *p* < 0.01) and negatively correlated with the fraction of clean energy (Cep) (Spearman's ρ = −0.645; p < 0.01) (Fig. 4a and Supplementary Material Table S10). These correlations highlight the significant contribution of fossil energy-based industrial emissions to legacy P accumulation by depositing P on various landscape surfaces, such as hardened pavements and soils [39]. Conversely, a negative relationship between legacy P and precipitation (Prec) (Spearman's *ρ* = −0.531; *p* < 0.01) indicates the pivotal role of precipitation-driven hydrological processes, such as surface runoff and soil erosion, in transporting legacy P from terrestrial to aquatic environments [31,45].

At the same time, drainage network coverage (Dra) was also negatively correlated with legacy P (Spearman's *ρ* = −0.925; *p* < 0.01). Precipitation, especially for short-term heavy-rainfall events, can result in CSOs. These events may bypass WWTPs, leading to the discharge of untreated sewage into rivers, which could potentially increase surface runoff and enhance riverine *LO*_P_, particularly in urban areas with dense populations and large sewer networks [46]. This underscores the critical role of drainage infrastructure in legacy P mobilization. In addition to rainfall events, climatic factors such as the frozen period (Froz) also play a role in legacy P dynamics, with a positive correlation (Spearman's *ρ* = 0.395; *p* < 0.05), which can be attributed to the fact that the prolonged frozen period in the SRB increases energy consumption for heating (Spearman's *ρ* = 0.559; *p* < 0.01), thus promoting fossil fuel use and associated P emissions [47]. These findings suggest that industrial emissions, drainage facilities, and climatic conditions, through their impact on fossil energy use and nutrient transportation, play a key role in shaping the dynamic trajectory of legacy P during the industrial dominance period.

As regional development shifted toward agriculture, cultivated soil properties, particularly black soil (Bsoil) and organic content (Orga), emerged as critical for legacy P accumulation (Spearman's *ρ* = 0.567 and 0.517, respectively; *p* < 0.01; Fig. 4b and Supplementary Material Table S10). Black soils, characterized by high organic content with abundant adsorptive active sites, have strong adsorption and retention capacities for soil P, making areas with prevalent black soils particularly susceptible to legacy P accumulation [29,48]. Conversely, the cultivated land slope (Slo) was negatively correlated with legacy P (Spearman's *ρ* = −0.646; *p* < 0.01), indicating that flatter terrains tend to reduce surface runoff and retain more legacy P in the soil [24,49].

Mulch film (Mulc), a common cultivation practice in cold regions, is positively associated with legacy P (Spearman's *ρ* = 0.547; *p* < 0.01), and can modify the soil environment to enhance soil P retention by improving moisture conservation, altering microbial activity, and maintaining organic balance [31,50]. Climatic conditions, especially the frozen period (Froz) and snow cover (Snow), showed positive correlations with legacy P (Spearman's *ρ* = 0.220 and 0.277, respectively; *p* < 0.05 and *p* < 0.01, respectively), which can be explained by soil hydrothermal movement.

Ice and snow cover, characterized by strong heat reflection and low thermal conductivity, drive the movement of dissolved P deeper into the soil profile by forming a surface insulating layer and creating a vertical temperature gradient that favors legacy P accumulation and prolongs the lag times [51]. These findings highlight the complex interplay between soil properties, cultivation practices, and climatic conditions in shaping the dynamic trajectory of legacy P during the agricultural dominance period.

#### 3.4.2 Strategic key points for controlling legacy P risks

Legacy risks, characterized by reserves of legacy P beyond natural uptake capacities and associated time lags, diminish the effectiveness of current reduction strategies [17,24]. The shift from industrial to agricultural dominance in the SRB led to a surge in NAPI and triggered profound changes in both agricultural and industrial practices, such as the widespread adoption of mulch film and agricultural irrigation, alongside modifications in industrial energy consumption and drainage infrastructure. This shift also resulted in the conversion of natural landscapes, such as forests and wetlands, into farmland [19,20,27,33]. These interrelated anthropogenic and natural factors collectively present the key and time-varying driving forces for controlling legacy risks.

**Fig. 5:**
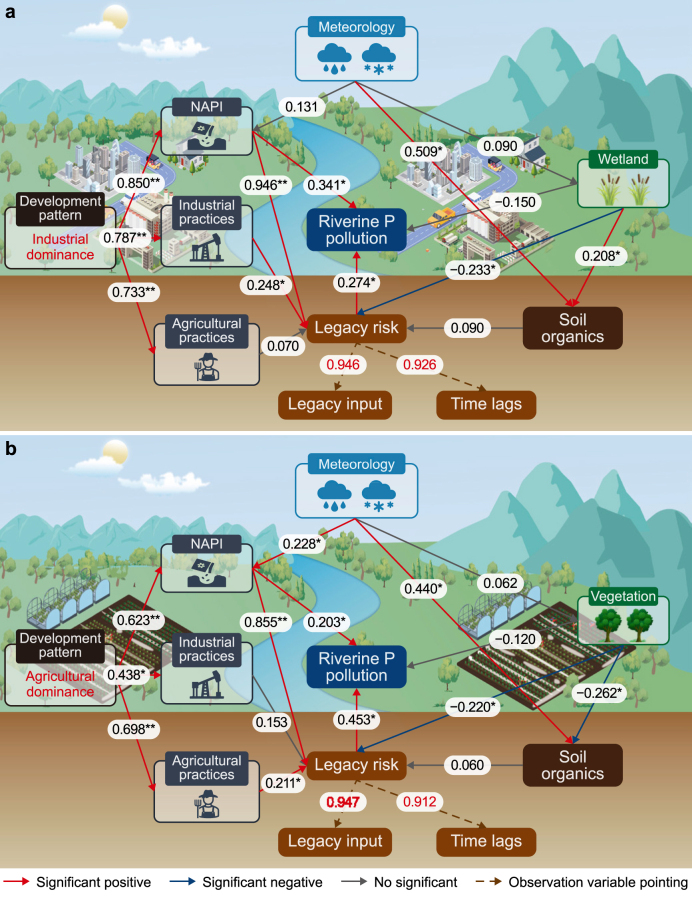
Partial least-squares path model (PLS-SEM; goodness of fit >0.6) revealed the integrated regulation of legacy risks and riverine P pollution by key factors before and after shifts in regional development patterns. **a**, PLS-SEM for the industrial development period from 1981 to 1990. **b**, PLS-SEM for the agricultural development period from 2010 to 2020. The number on the solid arrow is the path coefficient (*λ*), while the number on the dashed arrow represents the indicator loading; “∗” and “∗∗” denote significant effects with *p* < 0.05 and *p* < 0.01, respectively. NAPI: net anthropogenic P input.

The analysis, depicted in Fig. 5 and Table S14 (Supplementary Material), indicates that legacy risks are the dominant driver of riverine P pollution across the industrial (path coefficient *λ* = 0.274; *p* < 0.05) and agricultural (*λ* = 0.253; *p* < 0.05) dominance periods. Consistent with the results in section 3.3, the influence of legacy risks on riverine P pollution increased as regional development shifted from industrial to agricultural dominance, with the weight of *λ* (pointing to riverine P pollution) increasing from 44.5 % to 69.1 %. This increase in legacy P risk was attributed to the decreased P buffering capacity of the landscape (Supplementary Material Fig. S14) caused by the shift in development patterns in the SRB, which accelerated the mobilization and subsequent discharge of legacy P into the river system [10,24,26].

In terms of anthropogenic controls, NAPI played a pivotal role in legacy risks across both the industrial (*λ* = 0.946; *p* < 0.01) and agricultural (*λ* = 0.855; *p* < 0.01) dominance periods. However, the influence of NAPI on legacy risk decreased as the regional development focus shifted, with the weight of NAPI's *λ* among the anthropogenic drivers, including NAPI, agricultural practices, and industrial practices, decreasing from 75 % in the industrial period to 70 % in the agricultural period. This decrease was partly due to the increased significance of agricultural practices on legacy risks [31,40,50], with the weight of its *λ* on legacy risks increasing from 6 % to 17 % as developmental dominance shifted toward agriculture.

In terms of natural controls, wetlands were crucial for mitigating legacy P risks during the industrial dominance period, intercepting P runoff from landscapes (direct impact, *λ* = −0.233; *p* < 0.05) and enhancing soil organic conservation (indirect impact, *λ* = 0.208; *p* < 0.05) [17,27]. However, as the basin shifted toward agricultural development, the effectiveness of wetlands in mitigating legacy P risks diminished due to wetland degradation and changes in land use [19,20,33]. Concurrently, the role of vegetation in mitigating legacy P risks became more pronounced (*λ* = −0.22; *p* < 0.05) during the agricultural dominance period due to its capacity to phytosorb P from both soil-adsorbed and soil-solution forms [48]. Nevertheless, the increase in vegetation was associated with decreased soil moisture and consequent organic loss (*λ* = −0.262; *p* < 0.05), to some extent indirectly exacerbating legacy P risks to aquatic ecosystems (*λ* = 0.06) [20]. Thus, it is imperative to reassess and develop strategies to mitigate legacy P risks that are adapted to changing development patterns and from the perspective of anthropogenic and natural controls.

### 3.5 Future scenarios for legacy P risk mitigation by 2050

**Fig. 6:**
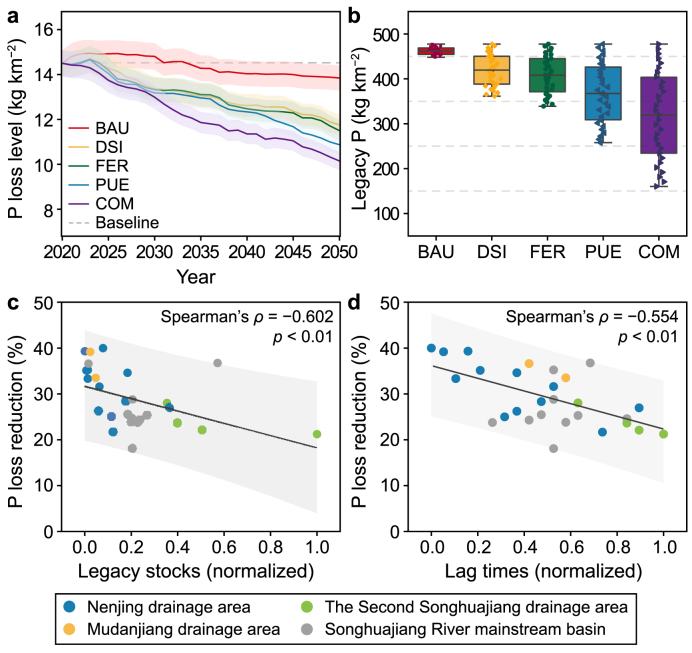
Mitigation of sustainable management strategies for P loss risk and legacy P pollution. **a**, P loss under various future scenarios from 2020 to 2050. **b**, Cumulative intensity of legacy P under various future scenarios by 2050. BAU: business-as-usual scenario; DSI: dietary structure improvement scenario; FER: fossil energy consumption reduction scenario; PUE: P use efficiency enhancement scenario; COM: combination scenario. **c**, Relationships between P loss reduction (from the baseline in 2020) and legacy stocks under the COM scenario by 2050. **d**, Relationships between P loss reduction (from the baseline in 2020) and lag times under the COM scenario by 2050.

With prospective scenarios, future trajectories of P loss (measured as the riverine P load at the basin outlet) and legacy P stocks up to 2050 were forecasted using an integrated machine learning model (Fig. 6; Supplementary Material Methodology S1.7). The following scenarios were used: (1) business-as-usual (BAU), (2) dietary structure improvement (DSI), (3) fossil energy consumption reduction (FER), (4) P use efficiency enhancement (PUE), and (5) combination (COM), which integrates all the above-optimized approaches. These scenarios were developed to provide insight into potential changes in P dynamics under different management strategies (detailed in Supplementary Materials Methodology S1.7 and Table S15).

Without any additional controls, the BAU scenario shows an initial increase in P loss during 2021–2026 due to legacy P influences, followed by an anticipated gradual decrease to a 4.5 % reduction in P loss by 2050 (Fig. 6a). This trend suggests that past reductions in NAPI (Fig. 2a) have already initiated the ongoing depletion of legacy P, which will continue over the next several decades, contributing to long-term P loss reductions. Under the BAU scenario, a projected decrease of 2.6 % in the annual legacy P stock by 2050 is anticipated (Fig. 6b). With the implementation of enhanced P management strategies, as outlined in the DSI, FER, and PUE scenarios, P loss reductions could be further enhanced, with anticipated decreases of 18.9 %, 20.7 %, and 25 %, respectively, by 2050 (Fig. 6a). Correspondingly, these strategies are also expected to reduce annual legacy P stocks by 24.3 %, 29 %, and 46 %, respectively, by 2050 (Fig. 6b). The COM scenario, which integrates all positive strategies, is predicted to achieve more substantial reductions in P loss and legacy P risks, with an estimated 30 % reduction in P loss and a 66 % reduction in annual legacy P stocks by 2050 (Fig. 6a and b). These results highlight the potential benefits of comprehensive P management strategies to simultaneously reduce P loss and mitigate legacy P pollution in the SRB (Supplementary Materials Fig. S17 and S18).

However, the effectiveness of P loss reduction under the same scenario exhibited considerable variation across the SRB subbasins (Fig. 6c and d). Notably, subbasins with lower extents of P loss reduction at outlets were associated with greater legacy P risks (i.e., greater legacy P stocks and time lags), with a statistically significant correlation (*p* < 0.01). Under the COM scenario, subbasins with lower legacy risks, for example, UN-1, GR, NMR, and UM in the Mudanjiang and Nenjiang drainage areas, are expected to achieve a reduction in P loss of approximately 33–40 % by 2050. Conversely, subbasins in the Second Songhuajiang drainage area, characterized by more pronounced legacy P risks, are expected to reduce P loss by only approximately 21–28 %. This finding underscores the ongoing challenge posed by legacy P risks in effectively controlling future P loss, exacerbated by the pronounced shift from industrial to agricultural dominance development patterns within the SRB.

### 3.6 Implications

The shift from industrial to agricultural development in the SRB since the 1980s (Fig. 1a) has significantly increased legacy P stocks and intensified pollution risks to aquatic environments (Fig. 1, Fig. 3a–b). This pivotal shift not only demands comprehensive reassessments of the sources and impacts of P loss but also necessitates strategic evolution in management paradigms to mitigate long-term environmental risks associated with legacy P effects [2,23].

First, sustainable and integrated management approaches for P legacies are urgently needed to align with shifts in regional development patterns. The exponential increase in legacy P requires management strategies that extend beyond the conventional focus on reducing industrial emissions to encompass the broader complexities of agricultural intensification [2]. Similar challenges have been observed in ecosystems such as Chesapeake Bay in the United States, where the composition of NAPI has significantly influenced the intensity of legacy P effects, with agricultural settings presenting more severe environmental risks than industrial contexts [52] (Fig. 3a). Historical data indicate that after 40 years of intensive cultivation and fertilizer application, the availability of P in black soil in the SRB decreased by 90.59 %, despite an increase in soil total P [53]. Innovative approaches, such as crop rotation, can significantly enhance soil organic matter and facilitate the conversion of total P into bioavailable forms [24]. In addition, reducing P fertilizer application following crop needs can promote crop uptake of legacy P without any loss in yield over extended periods [10,11,40]. It is estimated that the existing legacy P stock in the SRB could sustain crop production for 13 years without additional external P inputs (the P yield coefficient in harvested crops is 1100 kg P km^−2^ yr^−1^, and the average legacy P stock in the SRB is 14354.8 kg P km^−2^ yr^−1^) [2]. These strategies, coupled with dietary structure improvements, reductions in fossil energy consumption, and runoff control measures, can substantially mitigate legacy P accumulation and its adverse environmental impacts (Fig. 6b).

Second, the distinct spatial variability in legacy P risks across the SRB underscores the need for localized and differentiated management strategies. A significant disparity existed in the time lags between the P inputs and riverine P responses across the SRB (Fig. 3b), which can be attributed to the varied socioeconomic development and human activity intensity within the different subbasins. This variability mirrors global challenges, where local conditions significantly influence the efficacy of pollution management strategies [7,24]. Thus, flexible and tailored P management strategies could yield more effective outcomes. Our study demonstrates that applying uniform management strategies across all SRB subbasins could exacerbate legacy P risks inequalities by 2050 (Supplementary Material Fig. S18). Hence, targeted management strategies, especially for subbasins in the Second Songhuajiang drainage area with heightened legacy P risks, are crucial for improving water quality. By implementing these strategies, the SRB can serve as a model for sustainable P management in similar ecosystems worldwide, highlighting the indispensable role of adaptive management in tackling the complex challenges posed by legacy P amid shifting regional development patterns.

## 4 Conclusion

This study provides comprehensive evidence that shifts in regional development patterns from industrial to agricultural dominance have significantly exacerbated the risk of riverine pollution from legacy P in the SRB, based on a 40-year spatiotemporal dataset (1981–2020). The key findings are the following.

(1) Legacy P stocks were positively correlated with shifts in regional development patterns, which increased an astonishing 86-fold from 1981 to 2020, in sharp contrast to a 3.09-fold increase in net anthropogenic P inputs.
(2) With the shift from industrial to agricultural development, legacy P overtook current-year P inputs as the predominant source of riverine P changes, resulting in 4-to-23-year time-lagged responses in management effectiveness.
(3) In addition to developmental shifts, the impacts of fossil energy consumption and drainage management on legacy P dynamics have gradually diminished, while the influences of soil properties and cultivation practices have increased.
(4) Future scenarios suggest that combining strategic dietary adjustments, reduced fossil energy consumption, and improved agricultural P efficiency represents the best avenue for mitigating legacy P risk and basin P loss. However, subbasins with more pronounced developmental shifts will encounter greater challenges in mitigating legacy risks and restoring water quality.

Overall, these findings contribute to a better understanding of the intricate relationship between legacy P dynamics and rapid regional development change and highlight the importance of considering both development patterns and localized factors in developing effective strategies for sustainable P management.

## CRediT authorship contribution statement

**Wei Zhan:** Writing – original draft, Methodology, Investigation, Formal analysis, Data curation, Conceptualization. **Yedong Gao:** Methodology, Data curation. **Haoran Zhang:** Visualization, Software. **Yu Tian:** Supervision, Resources, Project administration, Funding acquisition, Conceptualization. **Yanan Zou:** Methodology. **Xiang Li:** Methodology. **Huihang Sun:** Software. **Lipin Li:** Supervision. **Yaruo Jin:** Data curation. **Jiaxin Cao:** Formal analysis. **Yiming Liu:** Formal analysis. **Nanqi Ren:** Supervision, Project administration.

## Declaration of competing interest

The authors declare that they have no known competing financial interests or personal relationships that could have appeared to influence the work reported in this paper.

Dr. Nanqi Ren, the Editor-in-Chief of *Environmental Science and Ecotechnology*, was not involved in the editorial review or the decision to publish this article.
