# Supplementary material for: Shifting regional development scenarios amplify legacy phosphorus threats to water quality
Source: Environ Sci Ecotechnol. 2025 May 9;26:100569. doi: 10.1016/j.ese.2025.100569 (PMC12141645; doi:10.1016/j.ese.2025.100569)
Supplement: Multimedia component 1 [file mmc1.docx]

Supplementary Material for

**Shifting Regional Development Scenarios Amplify Legacy Phosphorus Threats to Water Quality**

Wei Zhan ^a^, Yedong Gao ^a^, Haoran Zhang ^a^, Yu Tian ^a,^*, Yanan Zou ^b^, Xiang Li ^b^, Huihang Sun ^a^, Lipin Li ^a^, Yaruo Jin ^c^, Jiaxin Cao ^a^, Yiming Liu ^a^, Nanqi Ren ^a^

^a^ State Key Laboratory of Urban Water Resource and Environment (SKLUWRE), School of Environment, Harbin Institute of Technology, Harbin 150090, China

^b^ China Construction Power and Environment Engineering Co., Ltd., Nanjing 210012, China

^c^ Heilongjiang Institute of Energy and Environment, Harbin 150001, China

***Corresponding author:**

Yu Tian

State Key Laboratory of Urban Water Resource and Environment (SKLUWRE), School of Environment, Harbin Institute of Technology, Harbin 150090, China

E-mail: hit_tianyu@163.com

**This PDF file includes:**

Methodology (pages 1 to 10)

Figures and Tables (pages 11 to 43)

References (pages 44 to 46)

Data about annual NAPI and legacy P (Shown in Supplementary Data 1)

Data about GDP and ADP in 28 sub-basins (Shown in Supplementary Data 2)

**Methodology**

***Methodology S1.1 Study Area***

In this study, we applied a geographical information system (GIS) to define the basin boundary. The Songhuajiang River basin (SRB), located in Northeastern China (Figure S1). The SRB can be divided into four structural areas, namely the Changbai mountains (eastern part), the greater Khingan mountains (western part), the lesser Khingan mountains (northern part), and the Songnen plain (center and southern part) (Figure S1). The Songhuajiang (1900 km long) has two headstreams with the north source of the Nenjiang originating from the greater Khingan mountains and the south source of the 2^nd^ Songhuajiang from Tianchi Lake in the Changbai mountain (Figure S1). They flow together at Fuyu to form the Mainstream of Songhuajiang and joins the Amur River that eventually flows into the Sea of Okhotsk^1-3^.

Situated in the coldest region of China, the SRB belongs to a temperate humid and semi-humid continental monsoon climate zone with the monthly average temperature fluctuating from -19°C in January to 22°C in July^1^. The precipitation is mainly concentrated during July and August with an annual total of approximately 500 mm^2,3^. Despite the cold climate, paddy rice in the SRB is one of the most dominant agricultural crops, which attributed to its fertile black soils^1-3^. Moreover, soybean, corn, sorghum, and wheat are also the major cultivated crops in this basin. The main land cover types of the SRB include woodland, cropland, constructed land, wetland, and grassland (Figure S2). Moreover, 28 sub-basins within the SRB were divided based on the national third-level basin map. The spatial distribution and corresponding river of these sub-basins are showed in Figure S3. The full name, and corresponding hydrologic station and water quality section for each sub-basin are presented in Table S1.

***Methodology S1.2 Evaluation of Net Anthropogenic Phosphorus Input***

Net Anthropogenic Phosphorus Input (hereinafter abbreviated as NAPI; kg P/km²/yr) was calculated for the SRB. NAPI includes five major components: chemical fertilizer P application (CF), atmospheric P deposition (APD), seed P input (SI), non-food P input (NFI), and net food/feed P input (NFFI)^4^:

*NAPI = CF+APD+SI+NFI+NFFI* (S1)

At first, NAPI for the administrative regions within the SRB was estimated to address the subsequent subbasin-scale NAPI distribution. According to the administrative divisions in the SRB, the basin boundary is accurate to the district and county levels. Overall, an area-weighting method was used to extrapolate the P input from the administrative district-scale to the basin-scale. The sum of cropland and grassland area-weighting approach was used to calculate chemical fertilizer P for each sub-basin; cropland area-weighting approach was used for seed P, and impermeable land area-weighting approach was used for non-food P. For atmospheric P and net food/feed P input, we deem that the input of these P sources is not significantly affected by land cover types. Therefore, administrative district area-weighting approach was used for atmospheric P and net food/feed P input.

Data sources for estimating the NAPI of the SRB were mainly derived from statistical yearbooks that were downloaded mainly from China Economic and Social Data Research Platform (<http://data.cnki.net/YearData/Analysis>) and regional statistical yearbooks. For corresponding administrative regions, basic data (human and livestock populations, crop yields, chemical fertilizer use, cropland area, etc.) was collected at the provincial, city, and county levels. Other relevant data, such as atmospheric P deposition data, P content for crops and crop seeds, P consumption by human/livestock, were obtained from published literatures.

*Methodology S1.2.1 Fertilizer P application*

Annual chemical P fertilizer application amounts within the SRB were directly collected from China’s economic and social data research platform and Statistical Yearbooks of corresponding provinces and cities. According to previous studies, P fertilizer contains 12-18% of P_2_O_5_, and the total P fertilizer application is then converted to kg P by multiplying by 436.4 g P per kg P_2_O_5_ ^5, 6^.

*Methodology S1.2.2 Atmospheric P* *deposition*

Estimates of atmospheric P deposition were obtained from previous observations conducted for Eastern Asia over the 1954-2012 period^4, 7^. Here, linear interpolation was adopted to estimate atmospheric P deposition in the SRB from 1981 to 2012. Moreover, we found that atmospheric P deposition has a closed relationship with the consumption of fossil energy in the basin (R^2^ = 0.982; p<0.001) (Figure S4). Based on it, atmospheric P deposition within the basin during the 2013-2020 period was calculated and added as follows:

*P_dep_ = -8E-05×C _fossil energy_* *^2^ + 0.1795×C _fossil energy_ + 9.1361* (S2)

where *P_dep_* is atmospheric P deposition (kg/km^2^), *C _fossil energy_* is the consumption of fossil energy in the basin (standard coal/t).

*Methodology S1.2.3 Seed P input*

We chose vegetable and seven main agricultural crops to estimate seeding P in each research unit. The vegetable was represented by cabbage since the value of seeding P was slightly different between different kinds of vegetable. Seeding P per unit area for each crop type has been reported by Agricultural Technology Promotion Center of China (1999) ^5^. Seeding P was estimated by multiplying P input of seed per unit area for each crop type by cultivation area in each unit.

*Methodology S1.2.4 Non-food P*

Non-food P (NFI) was largely derived from the use of detergents in people's daily lives, and also included industrial P inputs from P mining, chemical P fertilizer production, and leakage from phosphate gypsum sites^5, 7^. NFI was generally estimated based on the population magnitude within a region, and the specific formula is as follows:

*NFI* = *POP* × *NIP / S*  (S3)

where *POP* is the population magnitude, *NIP* is the annual detergent consumption per capita (kg/capita/yr), *S* is the basin area (km^2^), and *PCD* is the P content in detergent (kg P /kg detergent)^5, 7^. Since 2002, the use of detergents with high P content has been prohibited in most areas of the SRB. According to the technical requirement for environmental labeling of products (detergents) in China (HJ 458-2009), we assumed a relatively high constant P content (i.e., 4.25%) in detergents before 2002 and a lower P content (i.e., 1.1%) after 2002^7^.

*Methodology S1.2.5 Net food/feed P input*

NFFI was calculated as the sum of human and livestock P consumption minus the sum of P in livestock and crop production^8^:

*NFFI* = *HC* + *AC* – (*G* – *GL*) – (*AP* – *APL*) (S4)

where *HC* is human consumption, *AC* is animal consumption, *G* is harvested grain, *GL* is grain loss due to pests, spoilage and processing, *AP* is animal products, and APL is spoilage and inedible animal products. The details for each P source calculation can be seen in Table S2-S4.

*Methodology S1.2.6* *Uncertainty analysis for NAPI* *estimations*

The Monte Carlo approach provides an estimation of uncertainty for the calculated values as it assumes that the uncertainty of the model inputs can be characterized by their statistical distribution functions^7^. It was assumed that all the parameters used in NAPI calculations followed a normal distribution with a variation coefficient of 0.3, which is widely applied in basin-scale nutrient budgeting researches^3^. The Monte Carlo sampling was used to randomly generate 10,000 sets of the parameters according to their normal distribution functions, resulting in 10,000 iterations to obtain the mean and 95% confidence interval for NAPI values. The Monte Carlo simulation was completed using a Crystal Ball software embedded in Microsoft Excel.

***Methodology S1.3 Cross-correlation analysis***

Cross-correlation analysis is a standard statistical method to measure correlation between two series of variables time-shifted against one another^9^. Currently, using this method to identify lags between input and output signals has become common in analysis of hydrologic or biogeochemical time series data^10^. Therefore, in this study, cross-correlation analysis was utilized to determine time lags between NAPI and riverine P loads at each sub-basin outlet. In this study, cross-correlation was evaluated over a long time of up to 40 years. The cross-correlation between the two variables is defined as follows:


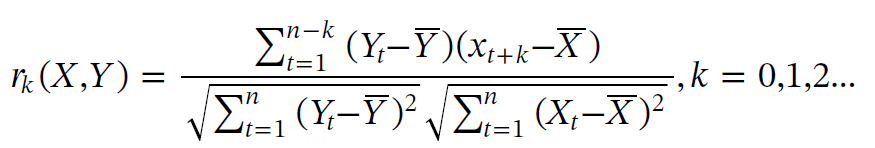


(S5)

where *Y_t_* refers to riverine P load in the *t*th year, *Y* is the average riverine P load over the entire time series, *X_t_* donates NAPI in the *t*th year, *X* is average NAPI over the entire time series, and *k* is an integer that denotes the lag interval (yr). The examination of cross-correlation was performed with the utilization of EViews software (Version 8).

***Methodology S1.4*** ***Geographical and temporal weighted regression***

Geographical and temporal weighted regression (GTWR) was applied to assess the contributions of P input sources to legacy P. GTWR is a regression method designed for elucidating the instability of correlation in time and space dimensions, which could interpret the spatiotemporal variability of the driving force explicitly^11^. First, we conducted a variance inflation factor (VIF) analysis of features (i.e., varied P input sources) in models. The calculation formula is as follows:

|  | $\text{VIF}\text{ = }\frac{\text{1}}{\text{1 –}\text{ }\text{R}_{\text{i}}^{\text{2}}}$ | (S6) |
| --- | --- | --- |

where *R_i_* is the negative correlation coefficient of the independent variable of other independent variables. The larger the VIF, the higher the collinearity, which could reduce the explainability of the model. Usually, a VIF of less than 10 for each feature is acceptable. Then the GTWR analysis realized through Eq (S5):

|  | $\text{y}_{\text{i}}\text{ }\text{=}{\text{ }\text{β}}_{\text{0}}\left( \text{u}_{\text{i}}\text{,}\text{v}_{\text{i}}\text{,}\text{t}_{\text{i}} \right)\text{ }\text{+}\text{ }\text{∑}\text{β}_{\text{k}}\left( \text{u}_{\text{i}}\text{,}\text{v}_{\text{i}}\text{,}\text{t}_{\text{i}} \right)\text{X}_{\text{ik}}\text{ }\text{+}\text{ }\text{ε}_{\text{i}}$ | (S7) |
| --- | --- | --- |

where u_i_, v_i_, and t_i_ represent the latitude coordinate, longitude coordinate, and sampling time of the sample point *i*; *y*_i_ represents the dependent variable value of the sample point *i*, and X_ik_ represents the kth independent variable of the sample point *i*; ε_i_ is the model error term; β_0_ (u_i_, v_i_, t_i_) and β_k_ (u_i_, v_i_, t_i_) are the regression constant and kth regression coefficient of the sample point *i*, respectively, and both are determined by the weight matrix of spatiotemporal position. As shown in Table S9, the VIF of selected features are in range of 1.4-5.7, greater lower than threshold of 10. This suggested that these features could be used for the next analysis. Such data processing was implemented using ArcMap 10.7 and the GTWR ADDIN V1.1.

***Methodology S1.5 Contribution analysis based on elasticity coefficient***

Contribution analysis based on the elasticity coefficient has been widely used to analyze the contributions of various factors influencing changes in the key behavioral factor index^3^. Here, the climate elasticity model was used to quantify the relative contributions of current-year P inputs and legacy P to changes in riverine P loads. The calculation steps of the attribution analysis based on the elasticity coefficient included: calculating the elasticity coefficient (ε_x_), calculating the influence (ΔQ_x_), and calculating the contribution (δQ_x_)^3^. The period from 1981 to 2020 was divided into four phases (i.e., 1981-1990, 1991-2000, 2001-2010, and 2011-2020) to analyze the interannual changes in the contribution of the factors.

(1) The elasticity coefficient ε_x_ is expressed as:


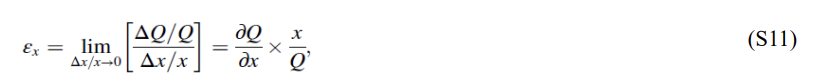
 (S8)

where Q is the riverine P loads, and x is the impact factor, including current-year P inputs and legacy P. The positive (negative) ε_x_ of the x factor indicates that an increase (decrease) in the x variable leads to an increase (decrease) in the riverine P loads.

(2) The influence calculation (ΔQ_x_, including ΔQ_cn_ and ΔQ_ln_) is performed to calculate the changes in riverine P loads caused by the change in each influence factor. The equation used to determine the effects of each factor on the changes in riverine P loads is as follows:


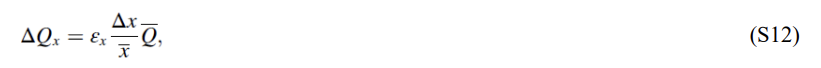
 (S9)

where Q is the multi-year average P load for the analysis period (i.e., 1981-1990, 1991-2000, 2001-2010, and 2011-2020), x is the corresponding multiyear average for the analysis period of the influencing factors, ε_x_ is the elasticity coefficient of each factor for the P load changes, and Δ_x_ is the difference between the average value of influencing factors during the analysis period and baseline (the average value of influencing factors in the first year and the two years before and after it) during the same period.

(3) The relative contribution (δQ_x_, including δQ_cn_ and δQ_ln_) is the percentage of the change caused by each factor in the total change of riverine P load. The relative contributions of current-year P inputs and legacy P to the changes in riverine P load can be obtained as follows:


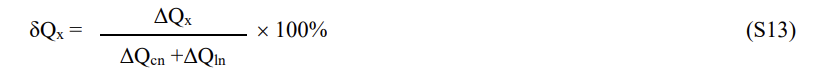
 (S10)

where ΔQ_x_ (including ΔQ_cn_ and ΔQ_ln_) is the variation of x (current-year P inputs and legacy P). ΔQ_cn_ +ΔQ_ln_ represents the total quantity value of riverine P load changes caused by the variations of each influence factor. Finally, the relative contributions of current-year P inputs and legacy P to the changes in riverine P load for the SRB are displayed in Fig.3c and d in main text.

***Methodology S1.6 Structural Model and Evaluation***

Partial least-squares path modeling (PLS-SEM) by SmartPLS (version 3.3.9) was performed to reveal the driving force of key factors on legacy and riverine P loss risks. Major structural model evaluation criteria are as follows:

1. R^2^ values of approximately 0.190, 0.333 and 0.670 are considered weak, moderate and substantial, respectively^12^.
2. Evaluate the path coefficient by p-value. Generally, there are statistical significance and highly statistical significance when P < 0.05 and P < 0.01, respectively.
3. The Goodness of Fit (GoF) criteria is used to assess the fitness of the SEM (GoF_small_ = 0.1, GoF_medium_ = 0.25, and GoF_large_ = 0.36)^13^.

According to the evaluation criteria of the measurement model and the path model, it could be seen that the SEM of this study fitted the data well in total. Specifically, the most of R^2^ values reached moderate or substantial levels (Table S13). And most of the p-values in the path coefficients were reached less than 0.05 (Table S14). For the complete model in early and later analysis periods, the GoF values were higher than 0.6, meaning that the models reached the GoF_large_ level. Moreover, we found that indicator loadings were higher than 0.9, satisfying the indicator reliability criteria^13^.

***Methodology S1.7 Random Forest model***

*Methodology S1.7.1 Model establishment*

In this study, the Random Forest model was applied to predict the P loss level and legacy P of each sub-basin across the SRB. To do so, the x-year moving average of P inputs, climate factors and land use indicators were selected as explanatory variables for modeling. The entire modeling procedure took place within the Python platform. The reasons for choosing these variables were as follows:

(1) X-year moving average of P input **(P-input)**.

The “x” is equal to magnitude of lag times for each sub-basin of the SRB. This paper suggested that for the SRB, long-term riverine P dynamics at any time point were not only influenced by current-years P input, but also historical P (legacy P) within the basin; such legacy P induced time-lags of riverine P responses to changes in basin P input (Fig.3a and b in main text). To reflect this lag-effect, hence, we utilized the moving averaged P input as an explanatory variable.

(2) Climate factors and land-use.

Previous studies indicated that riverine P trajectories are also affected by soil system characteristics and hydroclimate^14, 15^. The delivery of P sources to river system is mainly dependent on hydrological and biogeochemical processes within the basin^16^. The travel times of nutrients by surface runoff, soils, and groundwater to the river are on the order of days, years, and decades, respectively^17-19^. Incorporation of nutrients into soil organic matter and subsequent release of this P for potential leaching to the hydrosphere is estimated to require decades or even multi-decades^20, 21^. In this study, it was concluded that mulch film and drainage in soil system showed the key regulations to P accumulation and release, which was attributed to the agricultural development and the expanded cropland area. However, due to the unavailability of continuous relevant data for various landscapes, a combination of precipitation (prec), and cropland area percentage (crop) was adopted to represent the contribution of soil system due to their influence on soil mineralization and nutrient leaching. Moreover, precipitation could also reflect the contribution of hydrological factor. In addition, woodland (wood) area percentage was also selected as an explanatory variable owing to its increasing impact of plants to the P accumulation and release.

*Methodology S1.7.2 Future Simulations*

Future simulations for the 28 sub-basins within the SRB were simultaneously implemented. We conducted a scenario analysis to predict the change in P loss and legacy P reserves by 2050. The scenarios were set based on the global climate context in the future. The business-as-usual (BAU) scenario was set according to a moderate emission-based global climate context, SSP2-4.5, under which socio-economic factors follow its historical trend without significant changes (<https://www.resdc.cn>). Thus, in the BAU scenario, we assumed that there will be no variations in basin P management practices after 2020. The dietary structure improvement (DSI) scenario, fossil energy consumption reduction (FER) scenario, P use efficiency enhancement (PUE) scenario, and combination (COM) scenario were set based on a low emission-based global climate context, SSP1-2.6, which is simulated by the Integrated Assessment Model (IMAGE 3.0) (https://pcmdi.llnl.gov/CMIP6/). The characteristics of SSP1-2.6 mainly include: moderate population growth, technological progress in agricultural production, effective ecological environment restoration, mitigated extreme climate, and reduced consumption of animal products (<https://www.resdc.cn>). On other hand, given the trade-off between tree planting and wetland conservation (increasing afforestation has caused large loss in wetlands), a reasonable spatial optimization of future tree planting activities will be an urgent need to balance the carbon sequestration from forest gains and the protection of precious wetland resources especially in Northeast China^22^. Therefore, we assumed that in the future, woodland and wetland cover areas within the SRB had no significant changes under the spatial optimization for afforestation. Based on these two great backgrounds, we developed other specific P management strategies under the DSI, FER, PUE, and COM scenarios. Detailed information on the scenarios is showed in Table S15.

***Methodology S1.8 Long-term buffering index***

We estimated the long-term buffering index (LBI) for each watershed by determining the percentage of historically accumulated P that was being retained by a watershed in each study year^27^. The LBI value is an indication of the capacity of the watershed to absorb cumulative surplus P pressure and implies that some watersheds retain higher levels of accumulated P than others before releasing this P to a downstream water body. High LBI therefore signifies a large watershed buffering capacity, meaning that more P is retained in the watershed and therefore has a greater concentration of P per unit of watershed area. The LBI was calculated as the difference between cumulative NAPI and riverine P flux to the river in that study year:

$LBI=1-\frac{log({LO}_{P})}{log(cumulative NAPI)}$ (S11)

where *LO_P_* represents the annual riverine P load, i.e., Riverine TP flux (kg/km^2^/yr), *cumulative NAPI* was calculated the accumulated NAPI from 1981 to the study year (kg/km^2^/yr). The dynamics of LBI in the SBR are displayed in Fig.S14.

**Figures and Tables**


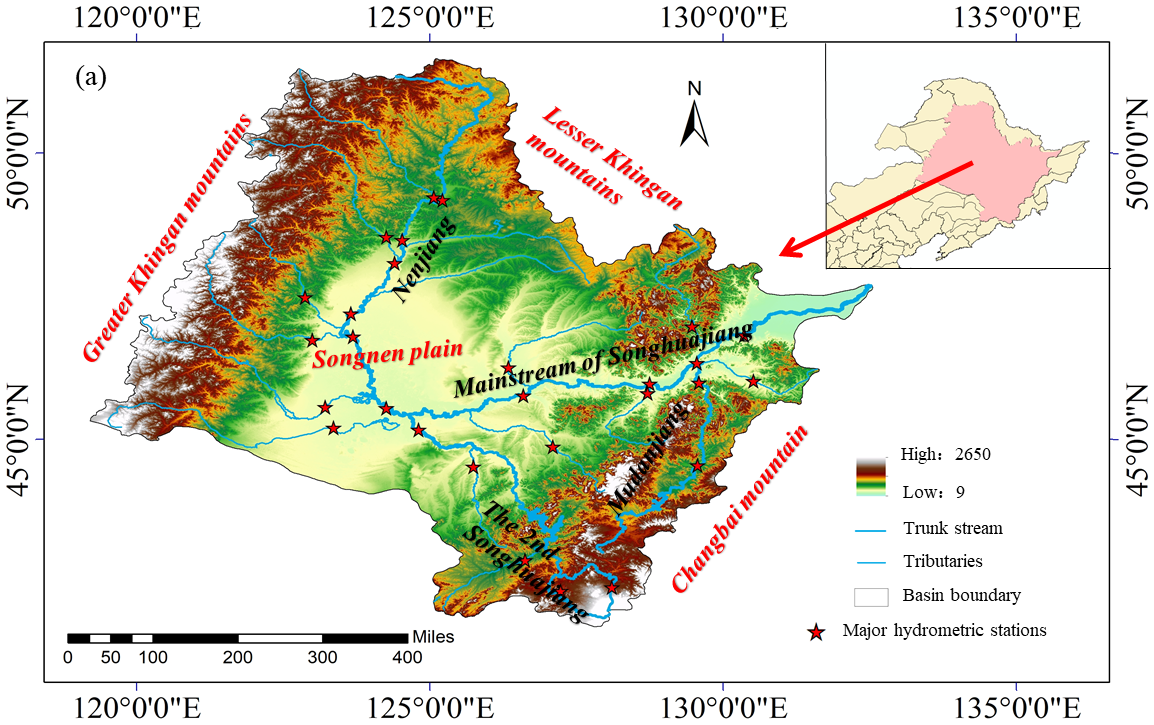


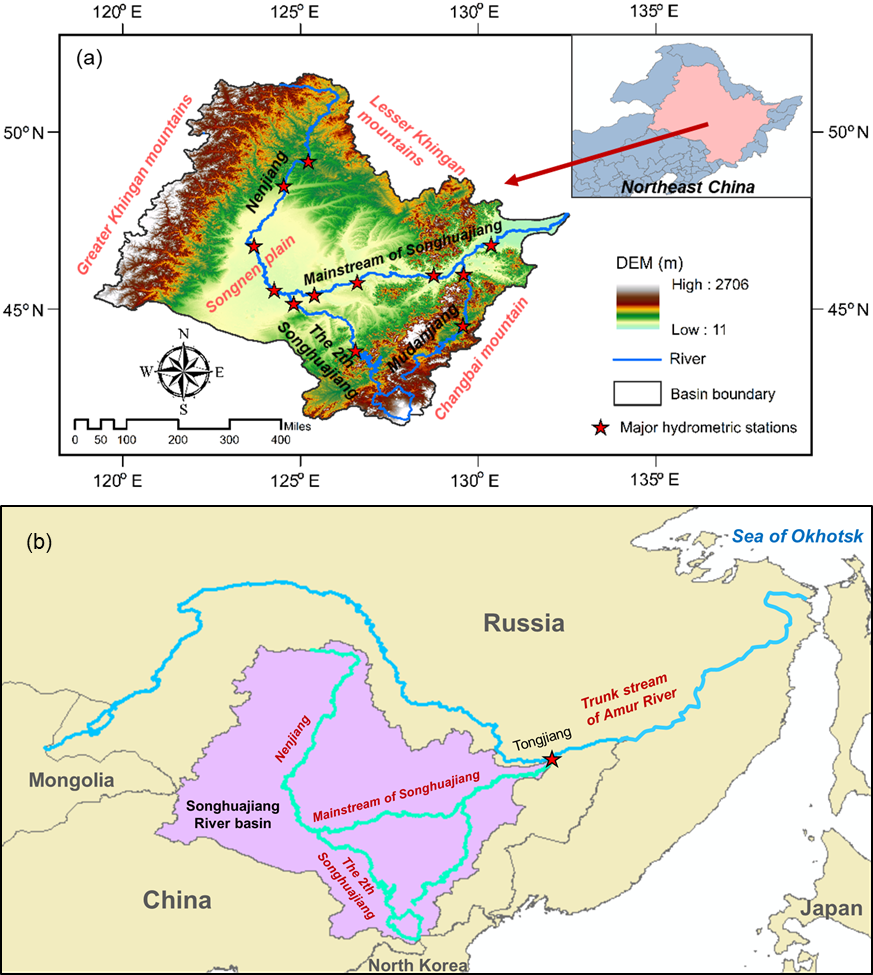


**Figure S1.** Songhuajiang River basin. (a) Basin location; (b) the end of Songhuajiang River.


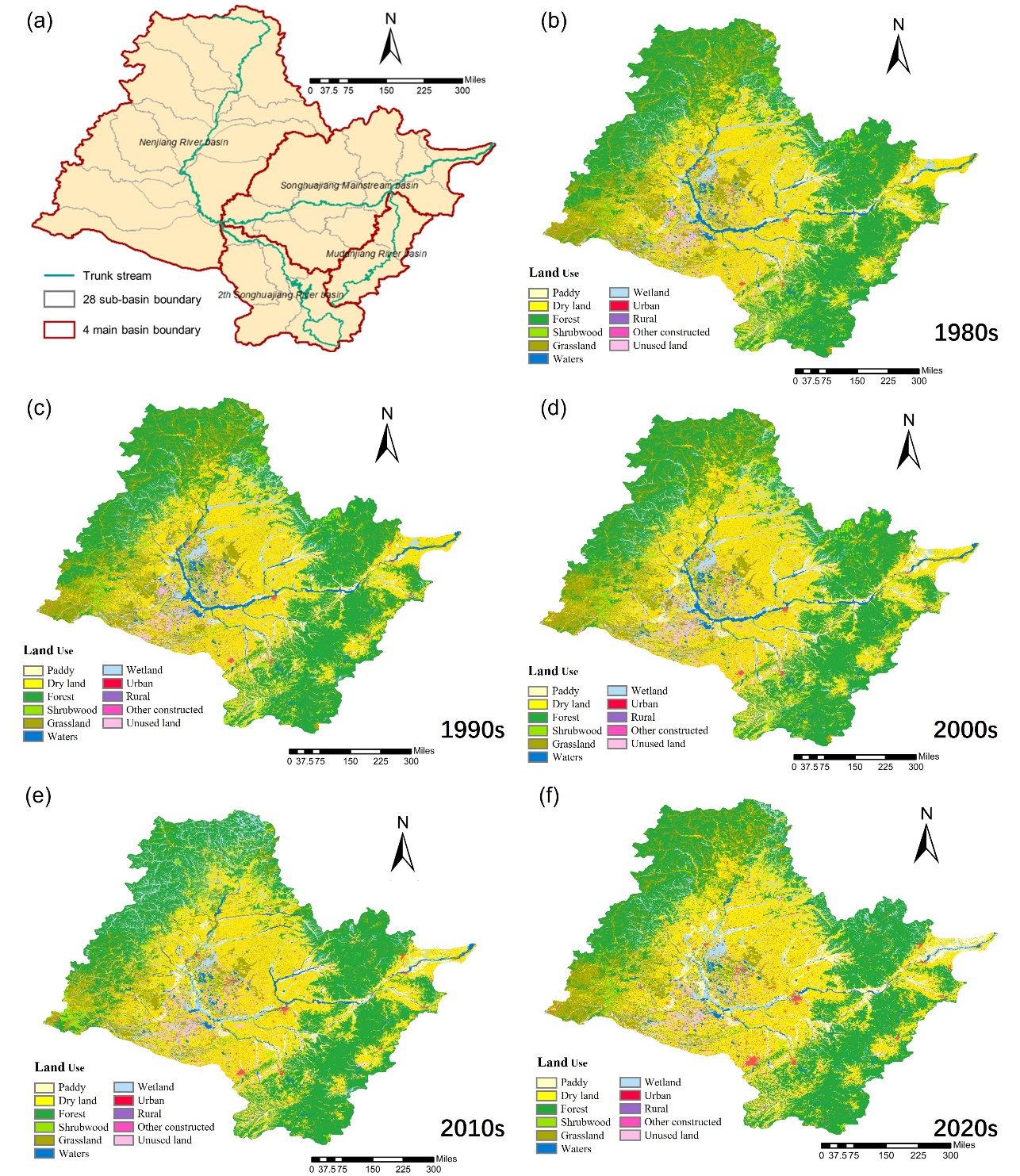


**Figure S2. Main streams and land-use cover in the Songhuajiang River basin.** (a) Distribution of main streams; (b) land-use cover in 1980s; (c) land-use cover in 1990s; (d) land-use cover in 2000s; (e) land-use cover in 2010s; and land-use cover in 2020s.


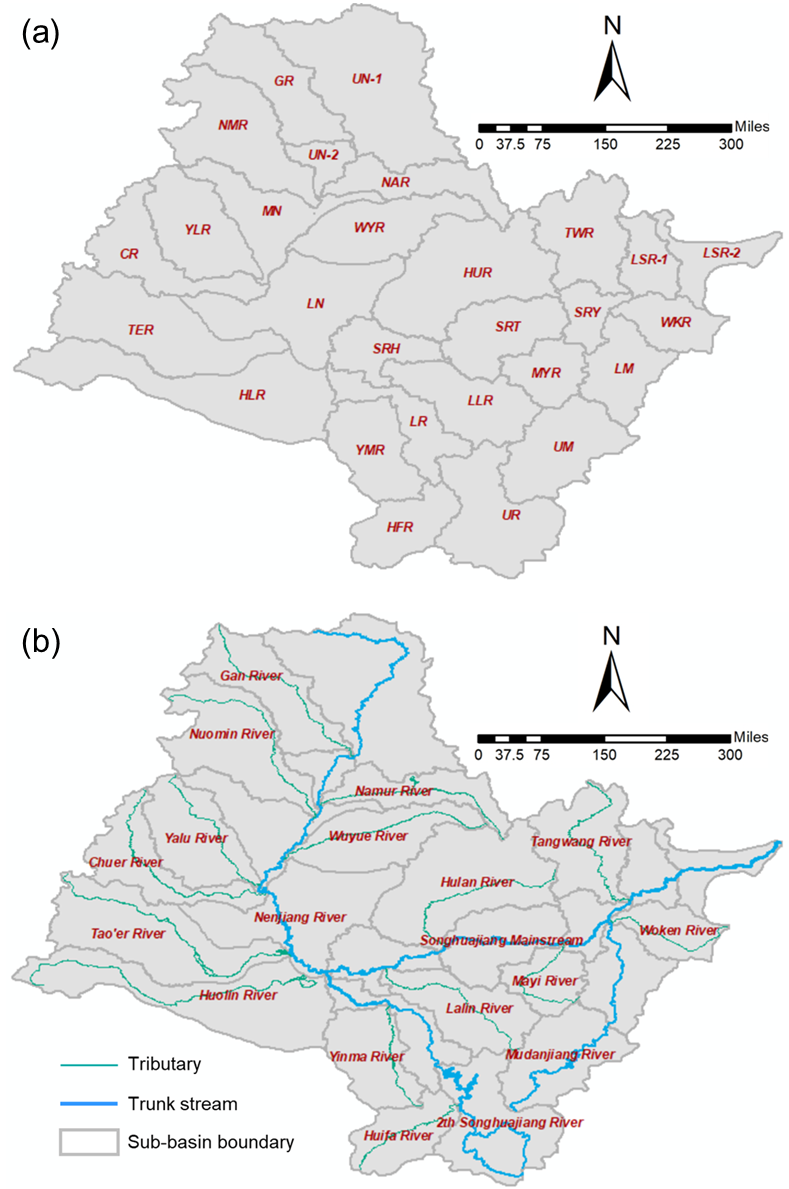


**Figure S3.** Spatial distribution (a) and corresponding river (b) of the sub-basins in the SRB.

**Figure S4**. Relationship between fossil energy consumption and atmospheric P deposition for the entire basin.


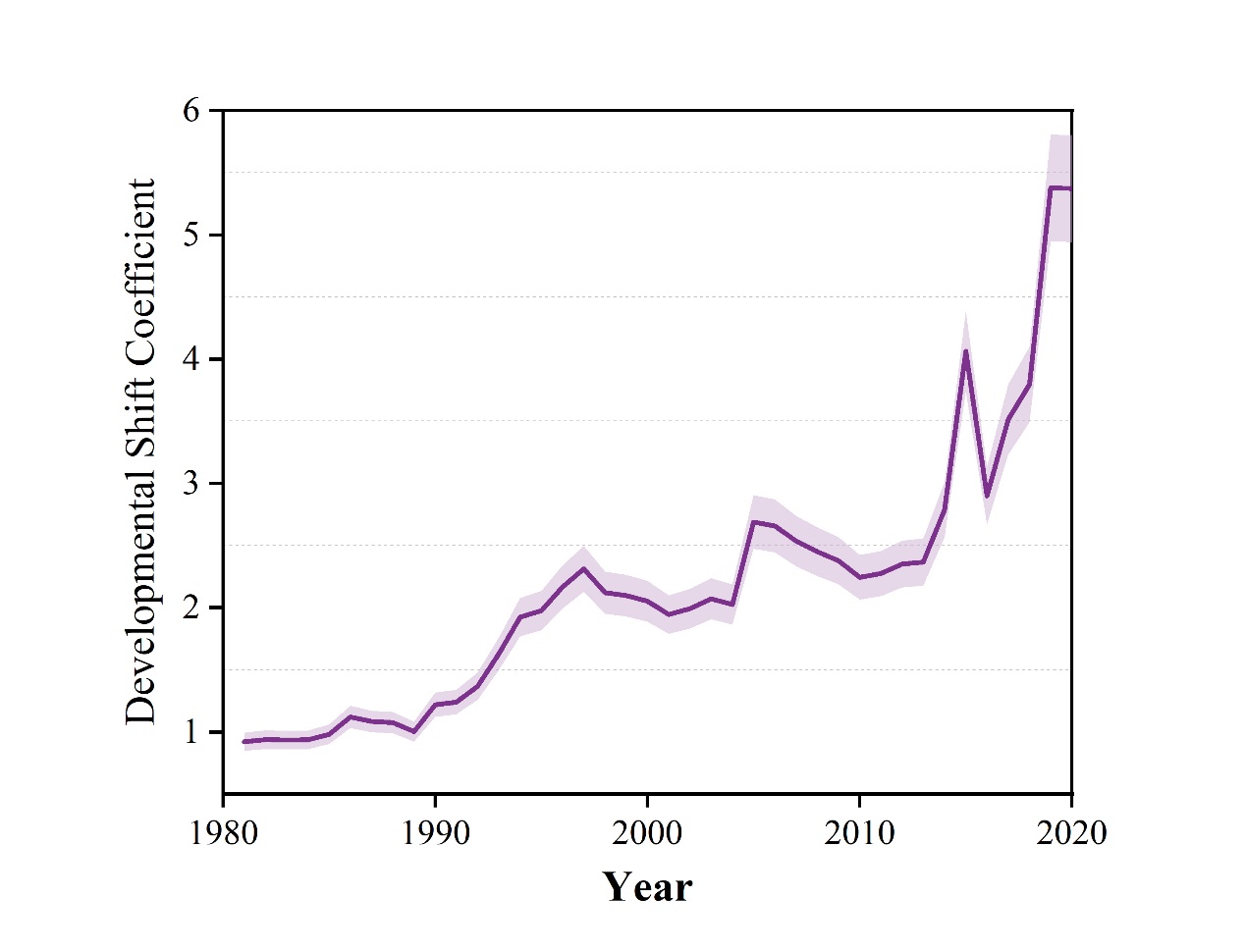


**Figure S5.** Development pattern shift for the entire basin.

**
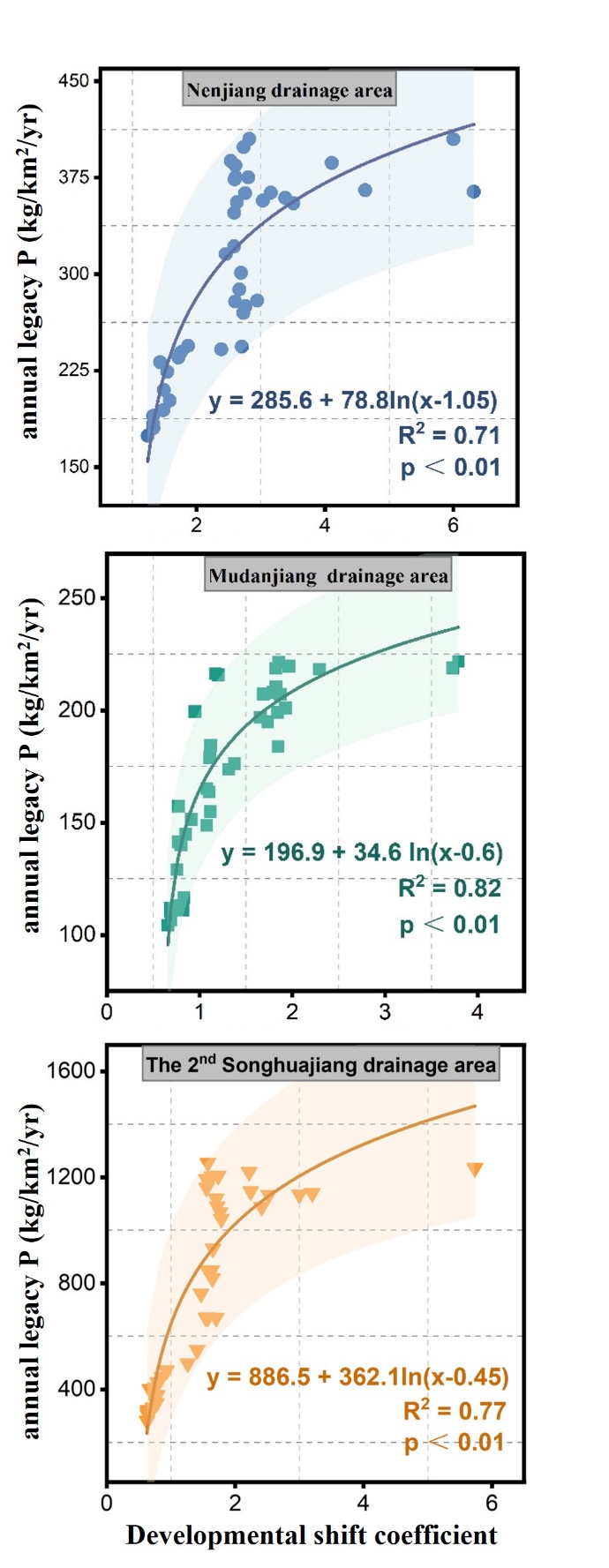
**

**Figure S6.** Relationship between annual legacy P and developmental shift coefficient for the main areas within the Songhuajiang River basin.

**
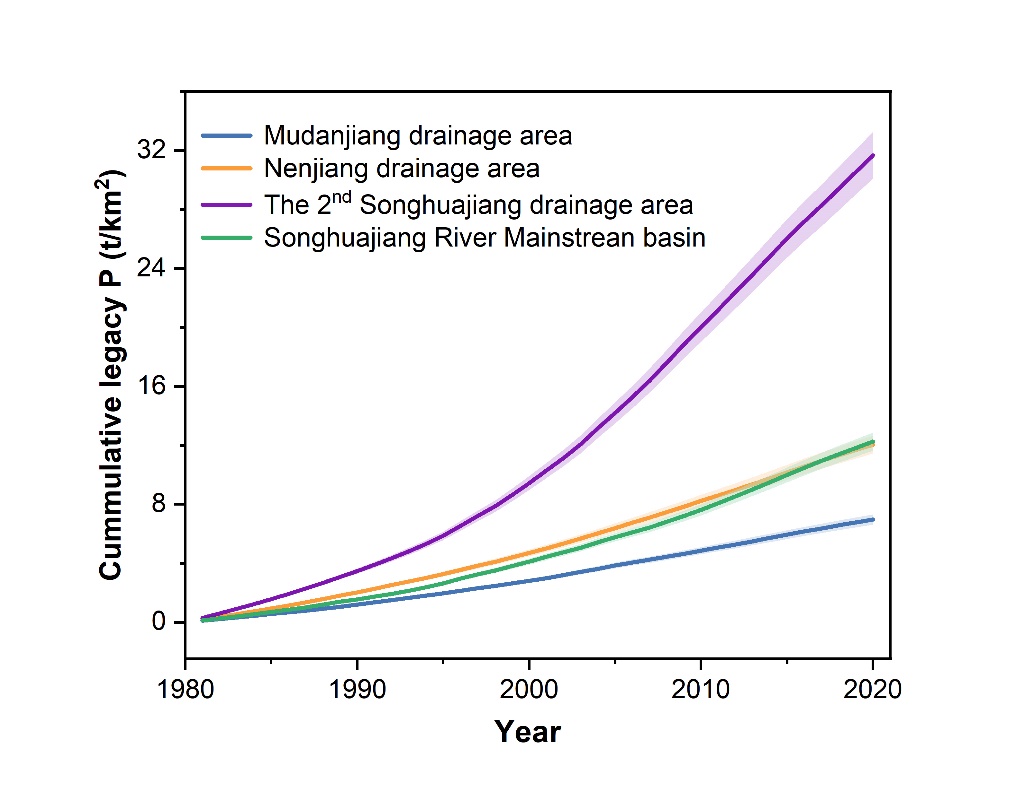
**

**Figure S7.** Accumulation of legacy P for the main areas within the Songhuajiang River basin during 1981-2020.

**
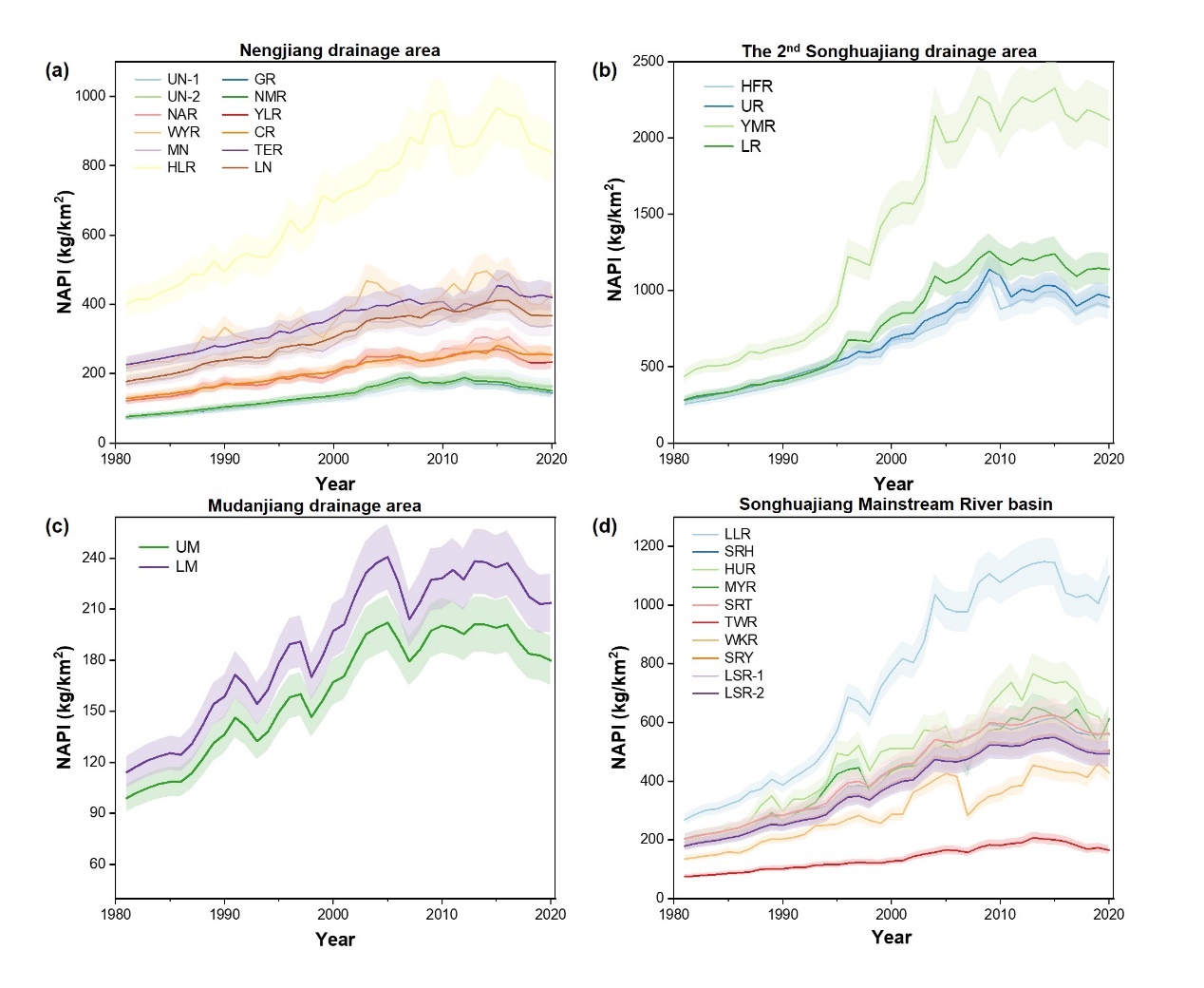
**

**Figure S8.** Historical trends of anthropogenic P sources over the 1981-2020 period for the 28 sub-basins of the Songhuajiang River basin (note different scales of y-axes).

**Figure S9.** Historical trend of net food/feed input components over the 1981-2020 period for the Songhuajiang River basin (f/f0 refers to the ratio between P sources in the ith year and the baseline 1981, the original data could be found in Table S5).

**
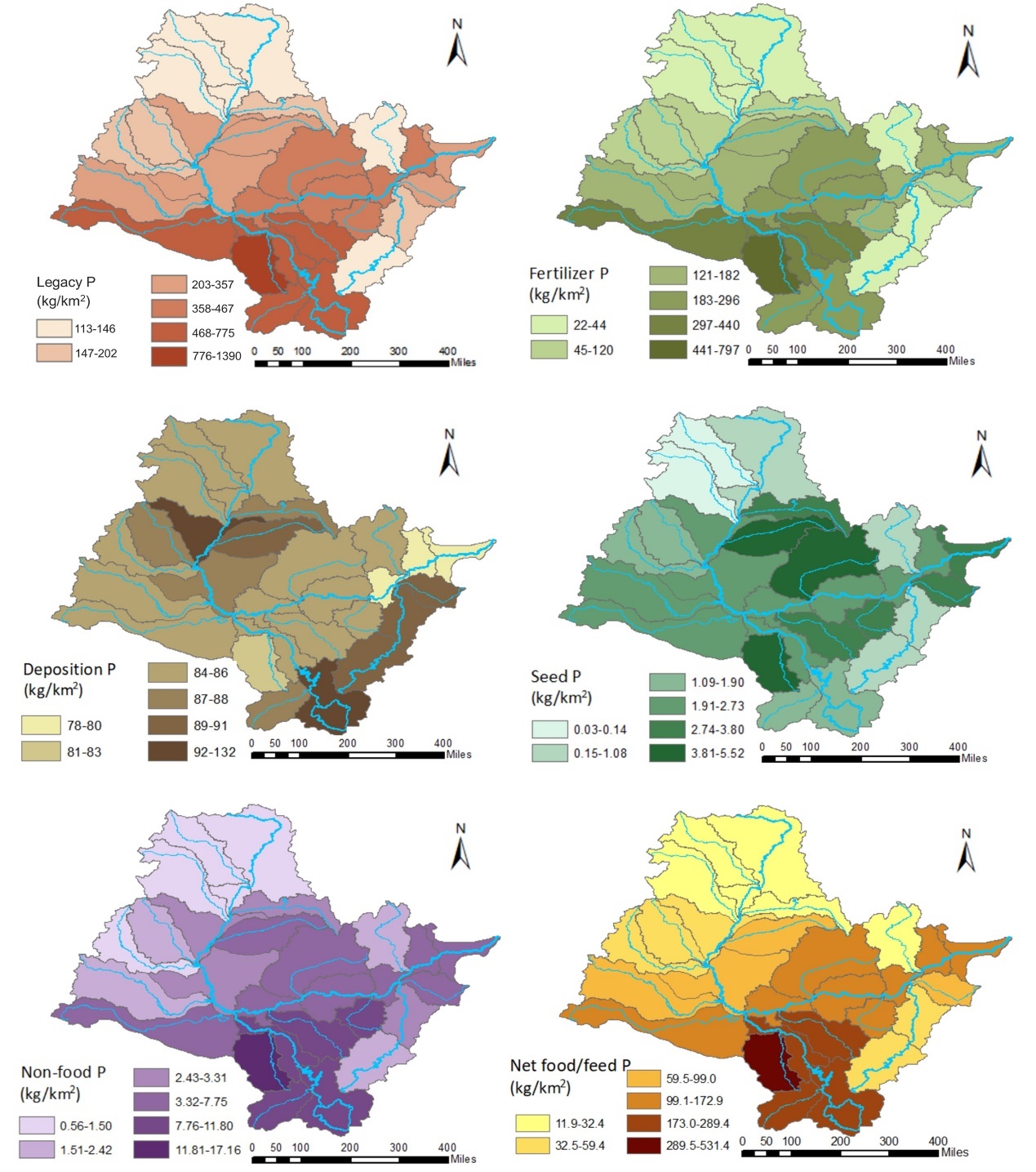
**

**Figure S10.** Spatial pattern of averaged legacy P and NAPI components (from 1981 to 2020).

**
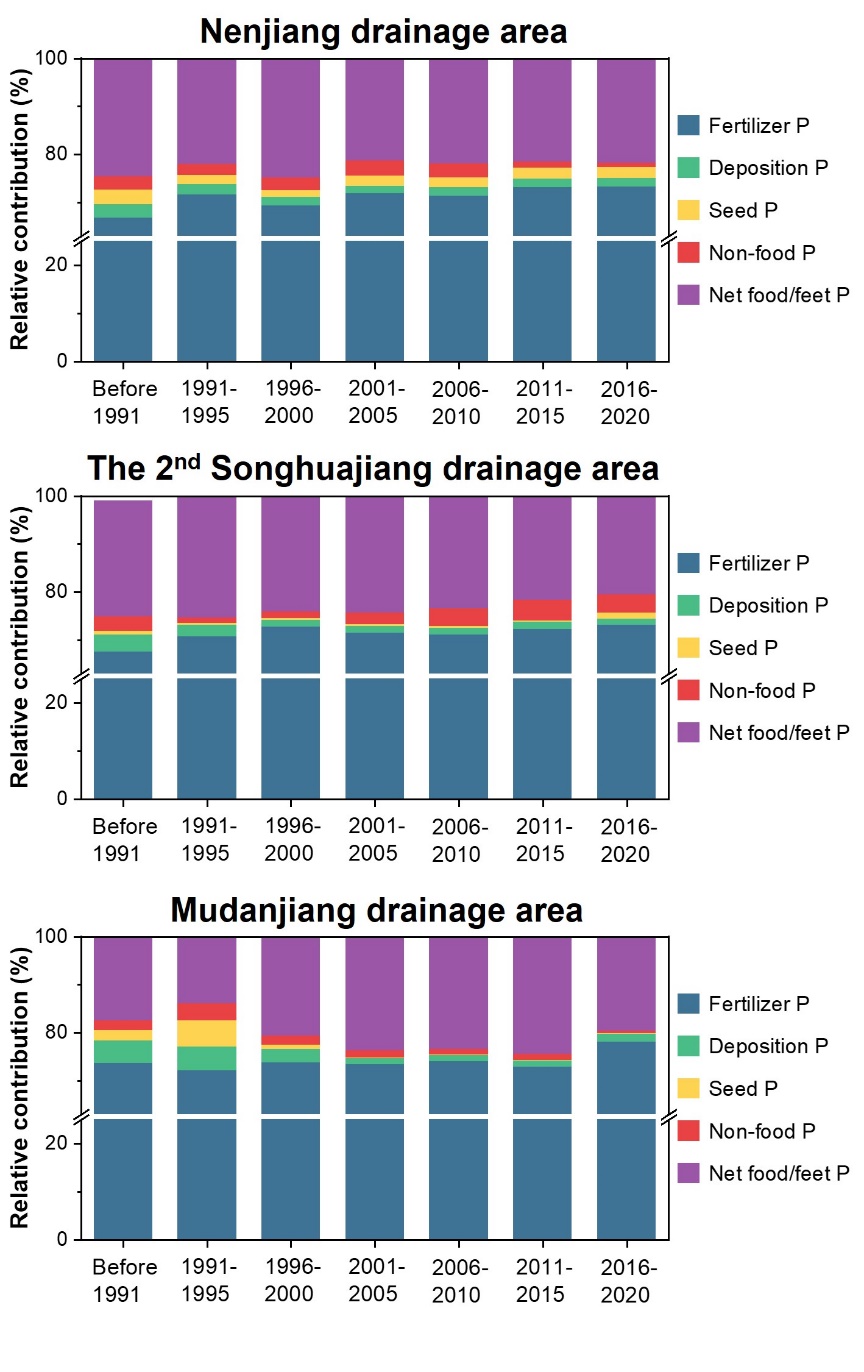
**

**Figure S11.** Contributions of anthropogenic P sources to legacy P for the main areas in the Songhuajiang River basin.

**
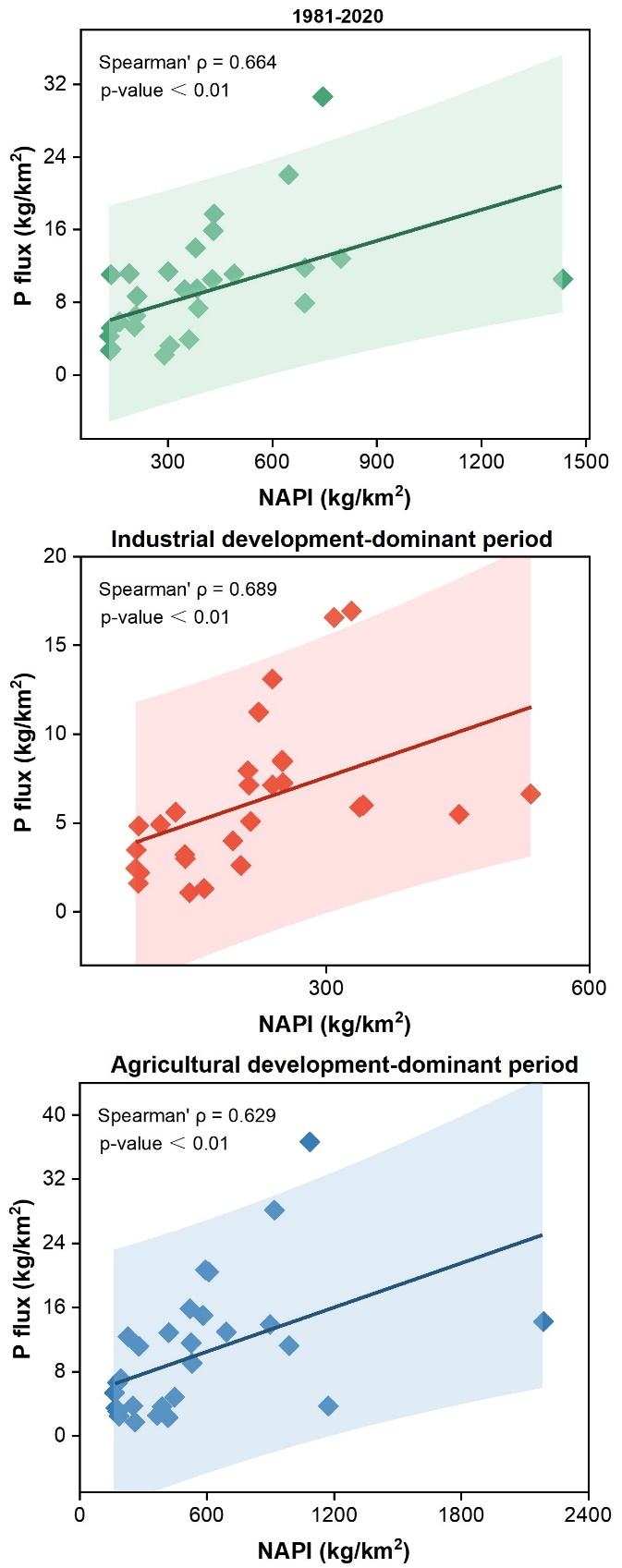
**

**Figure S12.** Relationships between NAPI and riverine P load (industrial development-dominant period refers to 1981-1990, while agricultural development-dominant period is presented as 2010-2020).

**
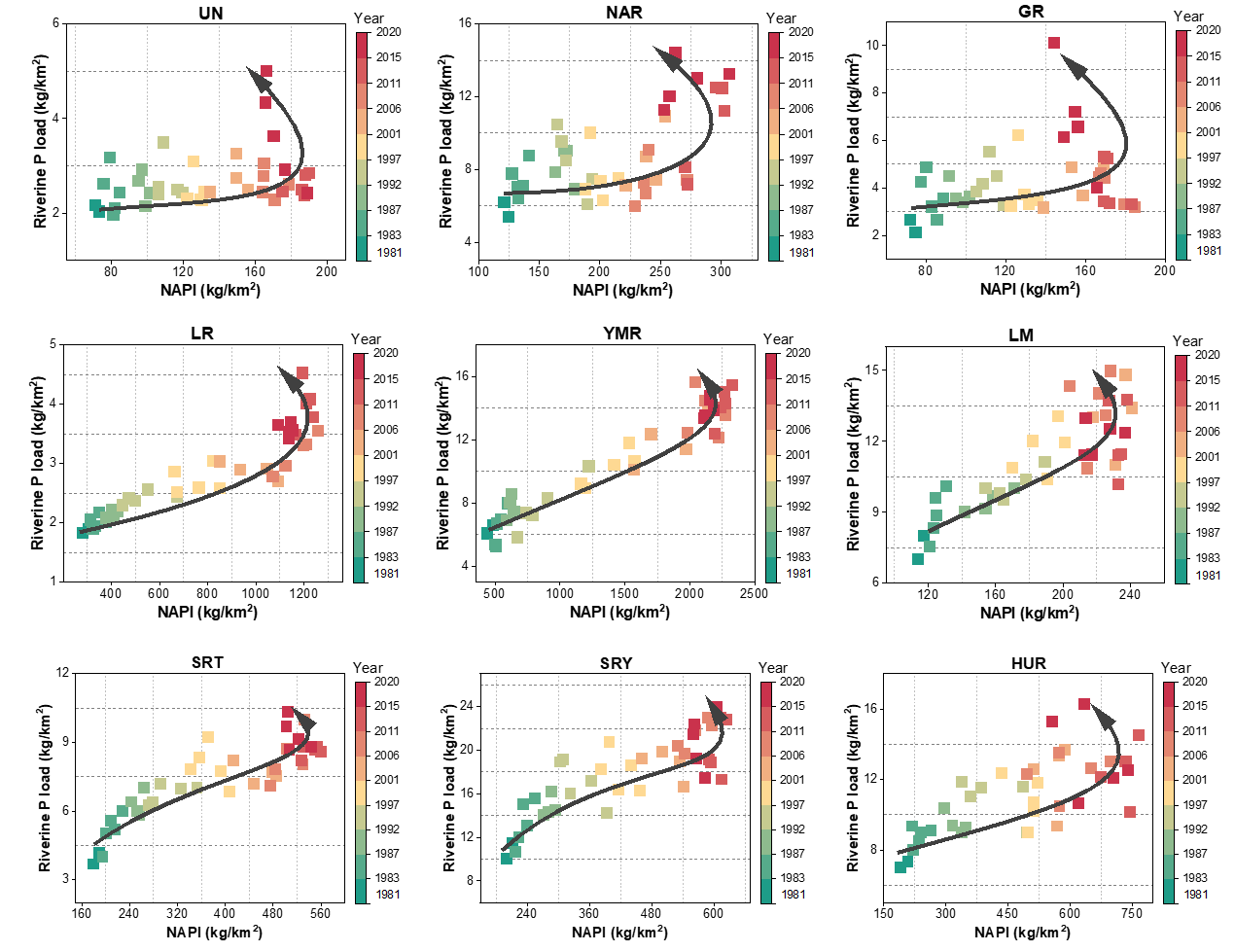
**

**Figure S13.** Time-varying relationships between NAPI and riverine P load for some typical sub-basins within the Songhuajiang River basin.

**
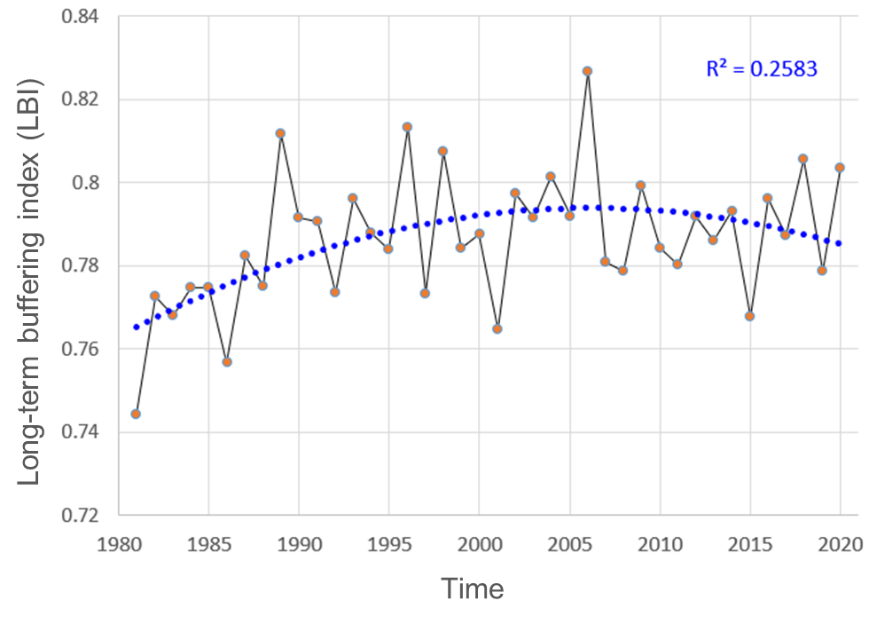
**

**Figure S14.** P buffering capacity (that is, to retain P in landscapes and to regulate the remobilization of P into water bodies) for the Songhuajiang River basin. As shown, LBI in the basin displayed a declined trend after the year 2006, indicating that the ability of the terrestrial ecosystem to retain accumulated legacy P had saturated, and more legacy P would release and transport into water bodies.


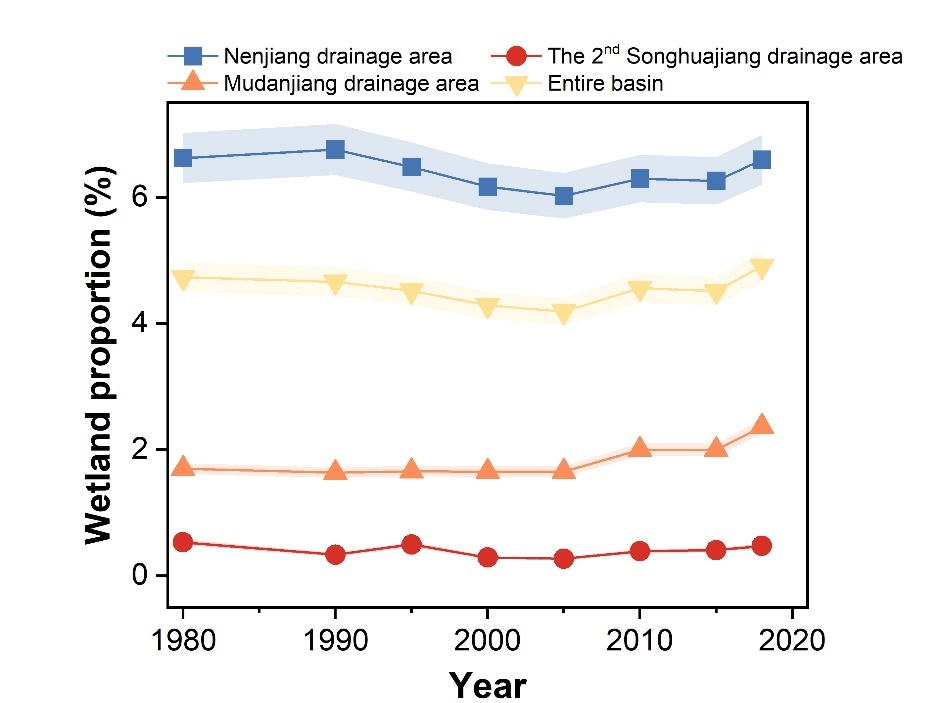


**Figure S15.** Changes of wetland cover proportions within the Songhuajiang River basin.

**
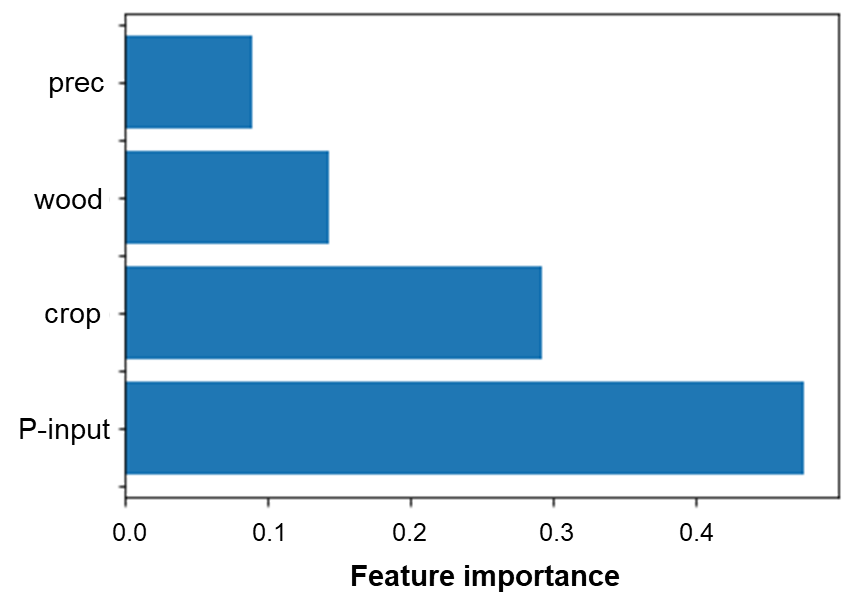
**

**Figure S16**. Feature importance for the Random Forest model (note: P-input, crop, wood, and prec refer to X-year moving average of P input, cropland area percentage, woodland area percentage, and precipitation, respectively).

**
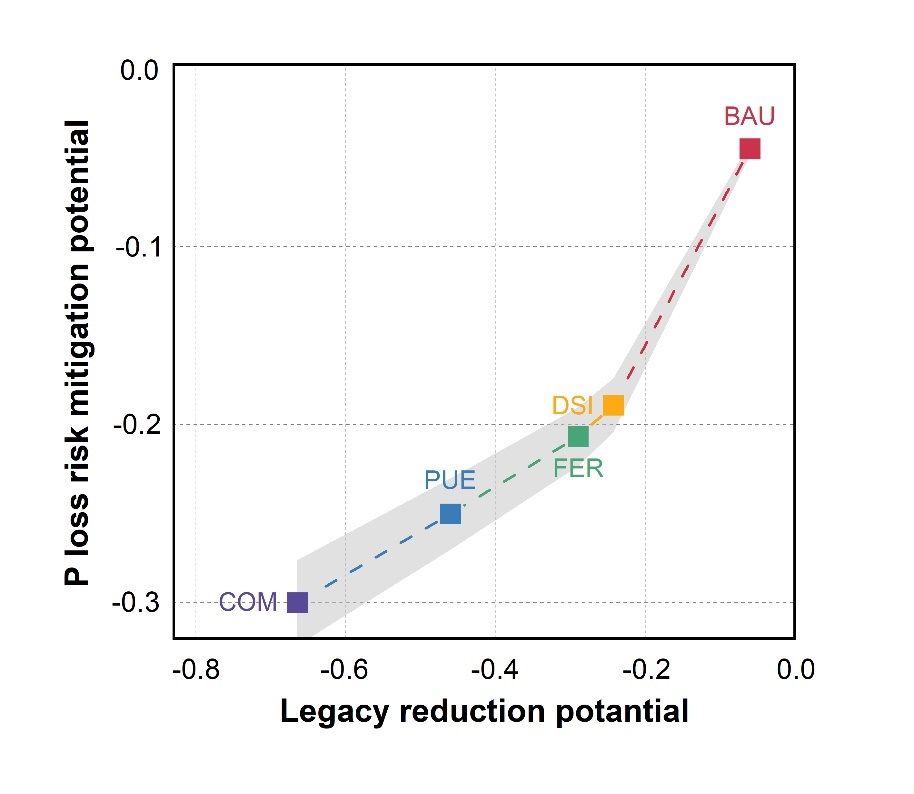
**

**Figure S17**. Relationships between legacy reduction and P loss mitigation potentials towards 2050 (note: negative sign in the figure represents reduction/mitigation).


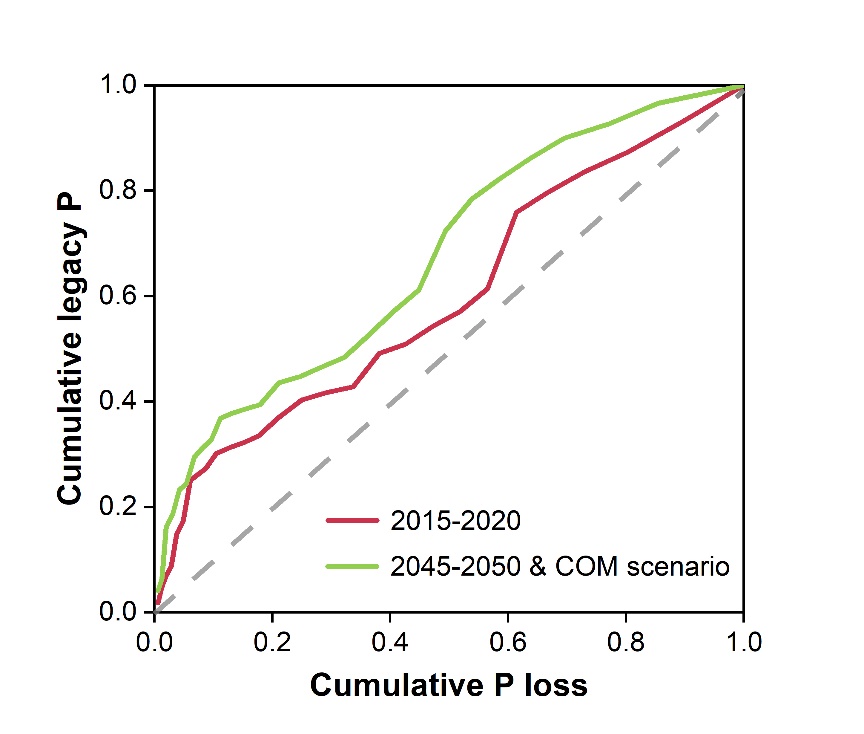


**Figure S18**. Cumulative probability of legacy P against cumulative probability of P loss (sorted by the increasing ratio of legacy P to P loss) in the current and future.

**Table S1.** Characteristics of the sub-basins within the Songhuajiang River basin.

| **Name** | **Drainage area** | **Abbreviation** | **Hydrologic station** | **Water quality section** |
| --- | --- | --- | --- | --- |
| Upper Nenjiang River-1 | Nenjiang drainage area | UN-1 | Nenjiang | Bohuotou |
| Gan River |  | GR | Liujiatun | Litun |
| Upper Nenjiang River-2 |  | UN-2 | Below Nierji | Fuyuancun |
| Namur River |  | NAR | Nahe | Namoer River Estuary |
| Nuomin River |  | NMR | Guchengzi | Chahayangxiang |
| Middle Nenjiang River |  | MN | Jiangqiao | Jiangqiao |
| Wuyue River |  | WYR | Yian Bridge | Liuyuan |
| Yalu River |  | YLR | Nianzishan | Genghis Khan |
| Chuer River |  | CR | Liangjiazi | Chuer River Estuary |
| Tao'er River |  | TER | Heidimiao | Daobao Bridge |
| Huolin River |  | HLR | Longzhao | Gaoliban |
| Lower Nenjiang River |  | LN | Dalai | Nenjiang River Estuary |
| Upper 2^nd^ Songhuajiang River | The 2^nd^ Songhuajiang  drainage area | USR | Jilin | Great Fengman |
| Huifa River |  | HFR | Wudaogou | Fuxing |
| Yinma River |  | YMR | Dehui | Liuzhentun |
| Lower 2^nd^ Songhuajiang River |  | LSR | Fuyu | Songlin |
| Upper Mudanjiang River | Mudanjiang drainage area | UM | Mudanjiang River | Hailang |
| Lower Mudanjiang River |  | LM | Changjiangtun | Mudanjiang River Estuary |
| Songhuajiang River to Harbin | Songhuajiang Mainstream River basin | SRH | Harbin | Zhushuntun |
| Lalin River |  | LLR | Wuchang | Miaojia |
| Hulan River |  | HUR | Lanxi | Hulan River Estuary |
| Songhuajiang River to Tonghe |  | SRT | Tong River | Baiduzhen |
| Mayi River |  | MYR | Lianhua | Mayi River Estuary |
| Songhuajiang River to Yilan |  | SRY | Yilan | Upper Jiamusi |
| Woken River |  | WKR | Woken | Woken River Estuary |
| Tangwang River |  | TWR | Chenming | Tangwang River Estuary |
| Lower Songhuajiang River-1 |  | LSR-1 | Jiamusi | Lower Jiamusi |
| Lower Songhuajiang River-2 |  | LSR-2 | Jiamusi | Tonghe |

**Table S2.** Data sources and parameters for calculating Net food/feed P.

|  | **Calculation** | **Parameters** | **References** |
| --- | --- | --- | --- |
| HC | Per capita phosphorus intake × Population | (Linear interpolation)  1980: 0.39 kg/capita  2002: 0.46 kg/capita  2009: 0.52 kg/capita | ^5^ |
| AC | First step:  calculating the average livestock population | *AL* (average animal population) =  *inventory*$\times\frac{1}{cycles}+\frac{sales}{cycles}\times\frac{cycles-1}{cycles}$ | ^7, 23^ |
|  | Second step:  P consumption × AL | See Table S3 | ^5-7^ |
| G | P content × Crop amount | See Table S4 | ^5, 7^ |
| AP | Production P × Livestock amounts | See Table S3 | ^5, 7^ |
| GL | = 0.1G |  | ^5, 7^ |
| APL | = 0.1AP |  | ^5, 7^ |

**Table S3**. Livestock P consumption and excretion rates.

| **Livestock type** | **P consumption (kg/cap/yr)** | **Production P (kg/cap/yr)** |
| --- | --- | --- |
| Pigs | 4.59 | 1.42 |
| Horses and cattle | 10.99 | 1.21 |
| Sheep | 1.26 | 0.2 |
| Poultry | 0.26 | 0.09 |
| Aquatic products | 2.16 | 1.60 |

**Table S4**. P content of agricultural crops.

| Crop type | P content (g/kg) |
| --- | --- |
| Rice | 1.10 |
| Corn | 2.44 |
| Wheat | 1.88 |
| Millet | 2.99 |
| Sorghum | 3.29 |
| Potato | 0.40 |
| Soybeans | 4.65 |
| Vegetable | 0.30 |
| Fruits | 0.13 |

**Table S5.** Human consumption, Livestock consumption, Livestock production and Crop production over the 1981-2020 period for the Songhuajiang River basin

| Type | Year | | | | | | | | | |
| --- | --- | --- | --- | --- | --- | --- | --- | --- | --- | --- |
| Human consumption  （kg/km^2^/yr） | **1981** | **1982** | **1983** | **1984** | **1985** | **1986** | **1987** | **1988** | **1989** | **1990** |
|  | 862.4 | 887.3 | 906.1 | 924.6 | 941.2 | 958.3 | 976.5 | 1006.8 | 1032.8 | 1050.5 |
|  | **1991** | **1992** | **1993** | **1994** | **1995** | **1996** | **1997** | **1998** | **1999** | **2000** |
|  | 1076.7 | 1093.3 | 1108.0 | 1124.5 | 1144.6 | 1162.7 | 1183.1 | 1197.5 | 1213.0 | 1235.8 |
|  | **2001** | **2002** | **2003** | **2004** | **2005** | **2006** | **2007** | **2008** | **2009** | **2010** |
|  | 1251.8 | 1268.2 | 1281.1 | 1298.5 | 1312.8 | 1331.2 | 1254.6 | 1368.3 | 1384.8 | 1396.7 |
|  | **2011** | **2012** | **2013** | **2014** | **2015** | **2016** | **2017** | **2018** | **2019** | **2020** |
|  | 1406.3 | 1406.0 | 1393.7 | 1378.8 | 1369.9 | 1360.6 | 1342.8 | 1339.0 | 1335.3 | 1262.1 |
| Livestock consumption  （kg/km^2^/yr） | **1981** | **1982** | **1983** | **1984** | **1985** | **1986** | **1987** | **1988** | **1989** | **1990** |
|  | 4235.7 | 4452.7 | 4684.7 | 4942.4 | 5200.1 | 5472.4 | 5749.9 | 5969.5 | 6306.5 | 6754.7 |
|  | **1991** | **1992** | **1993** | **1994** | **1995** | **1996** | **1997** | **1998** | **1999** | **2000** |
|  | 7283.4 | 7764.5 | 8048.2 | 9094.2 | 10235.2 | 11307.5 | 11904.1 | 11024.8 | 11843.2 | 12332.5 |
|  | **2001** | **2002** | **2003** | **2004** | **2005** | **2006** | **2007** | **2008** | **2009** | **2010** |
|  | 12857.7 | 13065.5 | 14348.5 | 16215.3 | 16870.1 | 15978.1 | 14854.1 | 18046.3 | 18528.5 | 19065.7 |
|  | **2011** | **2012** | **2013** | **2014** | **2015** | **2016** | **2017** | **2018** | **2019** | **2020** |
|  | 18264.1 | 18628.5 | 18705.5 | 18555.8 | 18473.9 | 16495.0 | 14859.8 | 14687.1 | 14815.8 | 15047.6 |
| Livestock production  （kg/km^2^/yr） | **1981** | **1982** | **1983** | **1984** | **1985** | **1986** | **1987** | **1988** | **1989** | **1990** |
|  | 856.2 | 887.9 | 907.9 | 937.9 | 978.1 | 1030.4 | 1066.1 | 1080.3 | 1139.1 | 1223.2 |
|  | **1991** | **1992** | **1993** | **1994** | **1995** | **1996** | **1997** | **1998** | **1999** | **2000** |
|  | 1296.0 | 1363.3 | 1409.4 | 1518.8 | 1674.8 | 1764.9 | 1859.9 | 1761.3 | 1862.2 | 1878.5 |
|  | **2001** | **2002** | **2003** | **2004** | **2005** | **2006** | **2007** | **2008** | **2009** | **2010** |
|  | 1933.1 | 2045.2 | 2207.5 | 2463.8 | 2634.7 | 2652.4 | 2394.0 | 2776.7 | 2899.1 | 3054.2 |
|  | **2011** | **2012** | **2013** | **2014** | **2015** | **2016** | **2017** | **2018** | **2019** | **2020** |
|  | 2751.6 | 2839.9 | 2858.2 | 2863.1 | 2817.8 | 2766.0 | 2973.1 | 2460.6 | 2526.5 | 2680.1 |
| Crop production  （kg/km^2^/yr） | **1981** | **1982** | **1983** | **1984** | **1985** | **1986** | **1987** | **1988** | **1989** | **1990** |
|  | 2007.5 | 2116.3 | 2256.9 | 2394.6 | 2560.0 | 2706.2 | 2822.8 | 2452.3 | 2389.9 | 3126.7 |
|  | **1991** | **1992** | **1993** | **1994** | **1995** | **1996** | **1997** | **1998** | **1999** | **2000** |
|  | 3352.1 | 3530.5 | 3832.1 | 4236.5 | 3989.2 | 4258.7 | 4711.3 | 4782.6 | 4743.8 | 3710.0 |
|  | **2001** | **2002** | **2003** | **2004** | **2005** | **2006** | **2007** | **2008** | **2009** | **2010** |
|  | 3797.0 | 4291.6 | 3899.3 | 5010.4 | 5651.6 | 5921.7 | 5303.5 | 7055.2 | 6847.2 | 7955.8 |
|  | **2011** | **2012** | **2013** | **2014** | **2015** | **2016** | **2017** | **2018** | **2019** | **2020** |
|  | 8333.3 | 9189.6 | 8571.7 | 8750.6 | 8786.0 | 8432.5 | 8223.6 | 8513.7 | 8831.2 | 9410.5 |

**Table S6.** Characteristic data of Songhuajiang River basin in 1981 and 2020

|  | **1981** | **2020** |
| --- | --- | --- |
| **Human population/10^4^** | 4566.2 | 4836.6 |
| **Grain acreage/ha** | 8832773.2 | 16509213.5 |
| **Livestock** | 122982175 | 370046126 |
| **Industrial enterprise** | 20980 | 5631 |
| **Land area/km^2^*10^4^** | 55.0 | 56.0 |

**Table S7.** Characteristic data of Sub-basins in the SRB in 1981

| **1981** | **Human population/10^4^** | **Grain acreage/ha** | **Livestock** | **Industrial enterprise** | **Land area/km^2^*10^4^** |
| --- | --- | --- | --- | --- | --- |
| **UN-1** | 54.1 | 345870.1 | 951116 | 162 | 54.1 |
| **NMR** | 20.6 | 102050.6 | 431112 | 45 | 2.6 |
| **GR** | 13.8 | 72372.4 | 263025 | 28 | 2.0 |
| **UN-2** | 12.4 | 49145.2 | 358956 | 38 | 0.5 |
| **NAR** | 73.0 | 300381.6 | 2131615 | 244 | 1.5 |
| **YLR** | 98.8 | 339481.4 | 3233954 | 304 | 3.0 |
| **LSR-2** | 61.7 | 217852.8 | 504103 | 347 | 1.0 |
| **MN** | 51.1 | 156499.9 | 1724020 | 168 | 0.6 |
| **LSR-1** | 59.9 | 219608.2 | 671954 | 346 | 0.9 |
| **WYR** | 371.1 | 866453.9 | 7332260 | 1766 | 3.5 |
| **TWR** | 59.3 | 103941.2 | 173403 | 175 | 2.0 |
| **CR** | 32.0 | 140665.4 | 1302670 | 84 | 1.8 |
| **HUR** | 314.5 | 713439.1 | 10105680 | 1205 | 3.0 |
| **LM** | 116.3 | 148992.9 | 2605799 | 575 | 1.5 |
| **TER** | 136.3 | 575695.3 | 5766588 | 406 | 4.5 |
| **WKR** | 114.0 | 243828.5 | 2543962 | 613 | 1.1 |
| **SRY** | 112.6 | 118474.1 | 3888591 | 505 | 0.7 |
| **SRT** | 343.7 | 337077.1 | 12399760 | 1559 | 1.7 |
| **LN** | 207.4 | 409013.5 | 4047622 | 1061 | 1.6 |
| **HLR** | 225.6 | 741372.8 | 9004532 | 735 | 3.5 |
| **LLR** | 409.7 | 475613.9 | 11685052 | 2038 | 2.0 |
| **SRH** | 177.5 | 342893.5 | 5491474 | 767 | 1.3 |
| **LR** | 348.8 | 476040.5 | 8406923 | 1716 | 2.1 |
| **MYR** | 207.5 | 203487.9 | 7485530 | 941 | 1.0 |
| **UM** | 115.2 | 153429.5 | 1302001 | 601 | 2.1 |
| **YMR** | 516.1 | 684079.6 | 11447908 | 2902 | 1.9 |
| **UR** | 121.8 | 103532.6 | 1203101 | 650 | 2.0 |
| **HFR** | 191.4 | 191479.9 | 6519465 | 999 | 1.4 |
| **Total** | 4566.2 | 8832773.2 | 122982175 | 20980 | 55.0 |

**Table S8.** Characteristic data of Sub-basins in the SRB in 2020

| **2020** | **Human population/10^4^** | **Grain acreage/ha** | **Livestock** | **Industrial enterprise** | **Land area/km^2^*10^4^** |
| --- | --- | --- | --- | --- | --- |
| **UN-1** | 57.1 | 666336.4 | 2457000 | 46 | 4.2 |
| **NMR** | 25.2 | 182516.3 | 1551964 | 16 | 2.6 |
| **GR** | 17.4 | 128313.6 | 1033120 | 11 | 2.0 |
| **UN-2** | 12.9 | 94295.1 | 907969 | 10 | 0.5 |
| **NAR** | 71.5 | 592015.0 | 4917725 | 63 | 1.5 |
| **YLR** | 101.9 | 650501.4 | 8258190 | 83 | 3.0 |
| **LSR-2** | 60.4 | 429400.7 | 2537773 | 89 | 1.0 |
| **MN** | 50.2 | 307860.9 | 4059901 | 43 | 0.6 |
| **LSR-1** | 58.7 | 432860.6 | 2916601 | 89 | 0.9 |
| **WYR** | 364.9 | 1699933.5 | 28600194 | 456 | 3.6 |
| **TWR** | 58.0 | 204874.1 | 4150658 | 45 | 2.1 |
| **CR** | 37.2 | 256236.4 | 4133925 | 28 | 1.8 |
| **HUR** | 307.9 | 1406230.4 | 31579505 | 311 | 3.1 |
| **LM** | 113.8 | 293673.7 | 5331340 | 148 | 1.5 |
| **TER** | 162.6 | 1014848.7 | 15464096 | 128 | 4.5 |
| **WKR** | 111.6 | 480600.4 | 6331727 | 158 | 1.1 |
| **SRY** | 110.3 | 233519.4 | 7773665 | 130 | 0.7 |
| **SRT** | 336.5 | 664398.7 | 23741714 | 402 | 1.8 |
| **LN** | 213.2 | 765911.5 | 17381763 | 278 | 1.7 |
| **HLR** | 264.4 | 1284422.0 | 16448634 | 213 | 3.5 |
| **LLR** | 437.4 | 869034.0 | 36126532 | 547 | 2.0 |
| **SRH** | 178.8 | 654568.4 | 15389215 | 200 | 1.4 |
| **LR** | 402.9 | 820293.5 | 31058274 | 475 | 2.1 |
| **MYR** | 203.1 | 401086.5 | 14332481 | 243 | 1.1 |
| **UM** | 120.8 | 288350.8 | 2850388 | 160 | 2.2 |
| **YMR** | 596.1 | 1178777.9 | 65186218 | 803 | 1.9 |
| **UR** | 140.7 | 178403.1 | 2958008 | 180 | 2.0 |
| **HFR** | 221.1 | 329950.4 | 12567549 | 276 | 1.4 |
| **Total** | 4836.6 | 16509213.5 | 370046127 | 5632 | 56.0 |

**Table S9**. Collinearity analysis for the features of the GTWR model.

| **Variables** | | **t-test** | **p-value** | **Collinearity statistics** | |
| --- | --- | --- | --- | --- | --- |
|  |  |  |  | **Tolerance** | **VIF** |
| Predictive | Legacy P | / | / | / | / |
| Explanatory | Chemical fertilizer | 387.765 | 0.000 | 0.190 | 5.273 |
|  | P deposition | 23.375 | 0.000 | 0.709 | 1.411 |
|  | Seed P | 3.866 | 0.000 | 0.696 | 1.436 |
|  | Non-food P | -3.199 | 0.001 | 0.174 | 5.735 |
|  | Net food/feed P | 192.180 | 0.000 | 0.374 | 2.671 |

**Table S10**. Spearman’s correlation coefficients and Mantel test to identify the key drivers for legacy P dynamics before and after shifts in regional development patterns across the Songhuajiang River basin. The correlation coefficient of legacy P dynamics in industrial development dominance was from the period 1981 to 1990, and that in agricultural development dominance was from the period 2010 to 2020. Red numbers represent significant correlations with p＜0.01, while bold numbers indicate correlations with p＜0.05.

| **Abbreviation** | **Full name** | **Industrial development** | | **Agricultural development** | |
| --- | --- | --- | --- | --- | --- |
|  |  | **Spearman’ ρ** | **Mantel’ r** | **Spearman’ ρ** | **Mantel’ r** |
| NAPI | NAPI | **0.686** | **0.427** | **0.998** | **0.731** |
| Indu | Industrial level | **0.917** | **0.596** | **0.567** | **0.171** |
| Agri | Agricultural level | **0.707** | 0.152 | **0.700** | **0.304** |
| Temp | Temperature | 0.263 | 0.033 | -0.359 | -0.055 |
| Prec | Precipitation | **-0.531** | **0.297** | -0.297 | -0.035 |
| Snow | Snow cover | 0.289 | **0.235** | **0.721** | **0.277** |
| Froz | Frozen period | **0.395** | 0.028 | **0.574** | **0.220** |
| Vege | Vegetation | -0.319 | -0.021 | **0.839** | **0.393** |
| Wetl | Wetland | **0.531** | **0.245** | 0.066 | **0.211** |
| Slo | Slope of cultivated lands | -0.138 | 0.057 | **-0.646** | **0.256** |
| Dry | Proportion of dry land area | 0.459 | 0.156 | **0.385** | **0.210** |
| Paddy | Proportion of paddy land area | 0.289 | **0.235** | **0.715** | 0.141 |
| Bsoil | Black soil | 0.105 | -0.050 | **0.567** | **0.171** |
| Orga | Soil organic content | 0.117 | -0.055 | **0.517** | **0.162** |
| Mulc | Mulch film | -0.358 | 0.087 | **0.547** | **0.290** |
| Dra | Drainage network coverage | **-0.925** | **0.639** | **-0.453** | 0.001 |
| Urb | Urbanization level | **-0.639** | **0.293** | -0.395 | 0.142 |
| Imper | Impermeable pavement | **-0.437** | -0.003 | -0.015 | -0.055 |
| Foss | Fossil energy consumption | **0.536** | -0.016 | 0.299 | 0.001 |
| Cep | Fraction of clean energy | **-0.645** | **0.434** | -0.389 | -0.022 |
| Sewa | Sewage treatment level | -0.240 | 0.009 | -0.160 | 0.068 |
| Flow | River flow | **-0.439** | **0.174** | -0.014 | -0.026 |

**Table S11.** The correlation between the drivers in industrial development dominance in the SRB.

| **Spearman’ρ** | **NAPI** | **Indu** | **Agri** | **Temp** | **Prec** | **Snow** | **Froz** | **Vege** | **Wetl** | **Slo** | **Dry** | **Paddy** | **Bsoil** | **Orga** | **Mulc** | **Dra** | **Urb** | **Imper** | **Foss** | **Cep** | **Sewa** | **Flow** |
| --- | --- | --- | --- | --- | --- | --- | --- | --- | --- | --- | --- | --- | --- | --- | --- | --- | --- | --- | --- | --- | --- | --- |
| **NAPI** | 1.000 | 0.617 | 0.664 | 0.224 | -0.814 | 0.269 | 0.621 | -0.384 | 0.814 | 0.088 | 0.929 | 0.269 | -0.015 | 0.001 | -0.449 | -0.772 | -0.527 | -0.506 | 0.504 | -0.685 | 0.062 | -0.583 |
| **Indu** | 0.617 | 1.000 | 0.758 | 0.395 | -0.475 | 0.236 | 0.326 | -0.317 | 0.475 | -0.123 | 0.396 | 0.236 | 0.211 | 0.200 | -0.378 | -0.909 | -0.614 | -0.452 | 0.475 | -0.654 | -0.277 | -0.507 |
| **Agri** | 0.664 | 0.758 | 1.000 | 0.327 | -0.471 | 0.220 | 0.343 | -0.452 | 0.471 | -0.128 | 0.547 | 0.220 | 0.252 | 0.262 | -0.238 | -0.716 | -0.350 | -0.534 | 0.372 | -0.511 | -0.234 | -0.443 |
| **Temp** | 0.224 | 0.395 | 0.327 | 1.000 | -0.176 | 0.543 | 0.309 | -0.134 | 0.176 | -0.331 | 0.030 | 0.543 | 0.283 | 0.270 | -0.057 | -0.372 | -0.054 | -0.003 | 0.345 | -0.174 | -0.369 | 0.189 |
| **Prec** | -0.814 | -0.475 | -0.471 | -0.176 | 1.000 | -0.111 | -0.479 | 0.304 | -1.000 | -0.287 | -0.787 | -0.111 | -0.011 | -0.008 | 0.311 | 0.627 | 0.437 | 0.378 | -0.337 | 0.427 | -0.163 | 0.379 |
| **Snow** | 0.269 | 0.236 | 0.220 | 0.543 | -0.111 | 1.000 | 0.169 | -0.082 | 0.111 | 0.031 | 0.190 | 1.000 | -0.054 | -0.050 | -0.126 | -0.347 | -0.037 | -0.139 | 0.249 | -0.173 | 0.045 | 0.112 |
| **Froz** | 0.621 | 0.326 | 0.343 | 0.309 | -0.479 | 0.169 | 1.000 | 0.354 | 0.479 | -0.074 | 0.544 | 0.169 | -0.093 | -0.092 | -0.534 | -0.357 | 0.108 | 0.280 | 0.559 | -0.145 | 0.122 | -0.002 |
| **Vege** | -0.384 | -0.317 | -0.452 | -0.134 | 0.304 | -0.082 | 0.354 | 1.000 | -0.304 | 0.094 | -0.335 | -0.082 | -0.403 | -0.440 | -0.163 | 0.415 | 0.624 | 0.930 | 0.089 | 0.540 | 0.158 | 0.545 |
| **Wetl** | 0.814 | 0.475 | 0.471 | 0.176 | -1.000 | 0.111 | 0.479 | -0.304 | 1.000 | 0.287 | 0.787 | 0.111 | 0.011 | 0.008 | -0.311 | -0.627 | -0.437 | -0.378 | 0.337 | -0.427 | 0.163 | -0.379 |
| **Slo** | 0.088 | -0.123 | -0.128 | -0.331 | -0.287 | 0.031 | -0.074 | 0.094 | 0.287 | 1.000 | 0.290 | 0.031 | -0.600 | -0.594 | -0.122 | -0.098 | -0.107 | 0.003 | 0.149 | 0.049 | 0.349 | -0.065 |
| **Dry** | 0.929 | 0.396 | 0.547 | 0.030 | -0.787 | 0.190 | 0.544 | -0.335 | 0.787 | 0.290 | 1.000 | 0.190 | -0.137 | -0.107 | -0.458 | -0.597 | -0.414 | -0.450 | 0.430 | -0.616 | 0.146 | -0.575 |
| **Paddy** | 0.269 | 0.236 | 0.220 | 0.543 | -0.111 | 1.000 | 0.169 | -0.082 | 0.111 | 0.031 | 0.190 | 1.000 | -0.054 | -0.050 | -0.126 | -0.347 | -0.037 | -0.139 | 0.249 | -0.173 | 0.045 | 0.112 |
| **Bsoil** | -0.015 | 0.211 | 0.252 | 0.283 | -0.011 | -0.054 | -0.093 | -0.403 | 0.011 | -0.600 | -0.137 | -0.054 | 1.000 | 0.989 | 0.170 | -0.015 | -0.104 | -0.243 | -0.224 | -0.221 | -0.415 | -0.158 |
| **Orga** | 0.001 | 0.200 | 0.262 | 0.270 | -0.008 | -0.050 | -0.092 | -0.440 | 0.008 | -0.594 | -0.107 | -0.050 | 0.989 | 1.000 | 0.152 | -0.018 | -0.118 | -0.274 | -0.227 | -0.228 | -0.400 | -0.175 |
| **Mulc** | -0.449 | -0.378 | -0.238 | -0.057 | 0.311 | -0.126 | -0.534 | -0.163 | -0.311 | -0.122 | -0.458 | -0.126 | 0.170 | 0.152 | 1.000 | 0.410 | 0.243 | -0.007 | -0.330 | 0.232 | -0.217 | 0.200 |
| **Dra** | -0.772 | -0.909 | -0.716 | -0.372 | 0.627 | -0.347 | -0.357 | 0.415 | -0.627 | -0.098 | -0.597 | -0.347 | -0.015 | -0.018 | 0.410 | 1.000 | 0.735 | 0.572 | -0.564 | 0.728 | 0.159 | 0.551 |
| **Urb** | -0.527 | -0.614 | -0.350 | -0.054 | 0.437 | -0.037 | 0.108 | 0.624 | -0.437 | -0.107 | -0.414 | -0.037 | -0.104 | -0.118 | 0.243 | 0.735 | 1.000 | 0.733 | -0.159 | 0.783 | 0.267 | 0.754 |
| **Imper** | -0.506 | -0.452 | -0.534 | -0.003 | 0.378 | -0.139 | 0.280 | 0.930 | -0.378 | 0.003 | -0.450 | -0.139 | -0.243 | -0.274 | -0.007 | 0.572 | 0.733 | 1.000 | -0.056 | 0.643 | 0.045 | 0.649 |
| **Foss** | 0.504 | 0.475 | 0.372 | 0.345 | -0.337 | 0.249 | 0.560 | 0.089 | 0.337 | 0.149 | 0.430 | 0.249 | -0.224 | -0.227 | -0.330 | -0.564 | -0.159 | -0.056 | 1.000 | -0.423 | 0.096 | -0.209 |
| **Cep** | -0.685 | -0.654 | -0.511 | -0.174 | 0.427 | -0.173 | -0.145 | 0.540 | -0.427 | 0.049 | -0.616 | -0.173 | -0.221 | -0.228 | 0.232 | 0.728 | 0.783 | 0.643 | -0.423 | 1.000 | 0.375 | 0.854 |
| **Sewa** | 0.062 | -0.277 | -0.234 | -0.369 | -0.163 | 0.045 | 0.122 | 0.158 | 0.163 | 0.349 | 0.146 | 0.045 | -0.415 | -0.400 | -0.217 | 0.159 | 0.267 | 0.045 | 0.096 | 0.375 | 1.000 | 0.214 |
| **Flow** | -0.583 | -0.507 | -0.443 | 0.189 | 0.379 | 0.112 | -0.002 | 0.545 | -0.379 | -0.065 | -0.575 | 0.112 | -0.158 | -0.175 | 0.200 | 0.551 | 0.754 | 0.649 | -0.209 | 0.854 | 0.214 | 1.000 |

**Table S12.** The correlation between the drivers in agricultural development dominance in the SRB.

| **Spearman’ρ** | **NAPI** | **Indu** | **Agri** | **Temp** | **Prec** | **Snow** | **Froz** | **Vege** | **Wetl** | **Slo** | **Dry** | **Paddy** | **Bsoil** | **Orga** | **Mulc** | **Dra** | **Urb** | **Imper** | **Foss** | **Cep** | **Sewa** | **Flow** |
| --- | --- | --- | --- | --- | --- | --- | --- | --- | --- | --- | --- | --- | --- | --- | --- | --- | --- | --- | --- | --- | --- | --- |
| **NAPI** | 1.000 | 0.586 | 0.691 | -0.351 | -0.291 | 0.713 | 0.568 | 0.841 | 0.083 | -0.631 | 0.384 | 0.710 | 0.586 | 0.535 | 0.552 | -0.449 | -0.384 | -0.024 | 0.298 | -0.387 | -0.142 | -0.014 |
| **Indu** | 0.586 | 1.000 | 0.489 | 0.084 | 0.005 | 0.404 | 0.251 | 0.656 | 0.161 | -0.211 | 0.500 | 0.322 | 1.000 | 0.943 | 0.406 | -0.289 | -0.371 | 0.341 | 0.565 | -0.184 | 0.387 | 0.066 |
| **Agri** | 0.691 | 0.489 | 1.000 | -0.245 | -0.152 | 0.863 | 0.444 | 0.819 | 0.113 | -0.631 | 0.514 | 0.539 | 0.489 | 0.466 | 0.627 | -0.481 | -0.523 | 0.114 | 0.562 | -0.341 | -0.160 | -0.083 |
| **Temp** | -0.351 | 0.084 | -0.245 | 1.000 | 0.372 | -0.115 | -0.383 | -0.270 | 0.228 | 0.582 | -0.082 | -0.241 | 0.084 | 0.157 | 0.204 | 0.577 | 0.355 | -0.131 | 0.021 | 0.589 | 0.211 | 0.080 |
| **Prec** | -0.291 | 0.005 | -0.152 | 0.372 | 1.000 | -0.102 | -0.047 | 0.031 | -0.019 | 0.280 | -0.109 | -0.519 | 0.005 | -0.105 | -0.133 | 0.392 | 0.334 | -0.175 | 0.174 | 0.538 | 0.482 | -0.177 |
| **Snow** | 0.713 | 0.404 | 0.863 | -0.115 | -0.102 | 1.000 | 0.444 | 0.790 | 0.070 | -0.483 | 0.364 | 0.550 | 0.404 | 0.394 | 0.643 | -0.378 | -0.330 | -0.124 | 0.309 | -0.258 | -0.125 | 0.068 |
| **Froz** | 0.568 | 0.251 | 0.444 | -0.383 | -0.047 | 0.444 | 1.000 | 0.635 | -0.064 | -0.630 | 0.348 | 0.587 | 0.251 | 0.181 | 0.191 | -0.690 | -0.258 | -0.027 | 0.314 | -0.634 | -0.112 | -0.010 |
| **Vege** | 0.841 | 0.656 | 0.819 | -0.270 | 0.031 | 0.790 | 0.635 | 1.000 | 0.133 | -0.710 | 0.557 | 0.565 | 0.656 | 0.574 | 0.600 | -0.560 | -0.463 | -0.009 | 0.543 | -0.403 | 0.059 | -0.135 |
| **Wetl** | 0.083 | 0.161 | 0.113 | 0.228 | -0.019 | 0.070 | -0.064 | 0.133 | 1.000 | 0.042 | -0.170 | 0.329 | 0.161 | 0.305 | 0.351 | -0.024 | 0.271 | -0.269 | -0.163 | -0.048 | -0.167 | -0.061 |
| **Slo** | -0.631 | -0.211 | -0.631 | 0.582 | 0.280 | -0.483 | -0.630 | -0.710 | 0.042 | 1.000 | -0.618 | -0.543 | -0.211 | -0.120 | -0.363 | 0.733 | 0.710 | -0.162 | -0.479 | 0.624 | 0.258 | 0.259 |
| **Dry** | 0.384 | 0.500 | 0.514 | -0.082 | -0.109 | 0.364 | 0.348 | 0.557 | -0.170 | -0.618 | 1.000 | 0.267 | 0.500 | 0.317 | 0.304 | -0.577 | -0.838 | 0.521 | 0.872 | -0.469 | 0.033 | -0.285 |
| **Paddy** | 0.710 | 0.322 | 0.539 | -0.241 | -0.519 | 0.550 | 0.587 | 0.565 | 0.329 | -0.543 | 0.267 | 1.000 | 0.322 | 0.358 | 0.531 | -0.618 | -0.307 | 0.083 | 0.139 | -0.593 | -0.424 | -0.136 |
| **Bsoil** | 0.586 | 1.000 | 0.489 | 0.084 | 0.005 | 0.404 | 0.251 | 0.656 | 0.161 | -0.211 | 0.500 | 0.322 | 1.000 | 0.943 | 0.406 | -0.289 | -0.371 | 0.341 | 0.565 | -0.184 | 0.387 | 0.066 |
| **Orga** | 0.535 | 0.943 | 0.466 | 0.157 | -0.105 | 0.394 | 0.181 | 0.574 | 0.305 | -0.120 | 0.317 | 0.358 | 0.943 | 1.000 | 0.478 | -0.201 | -0.227 | 0.225 | 0.380 | -0.132 | 0.311 | 0.157 |
| **Mulc** | 0.552 | 0.406 | 0.627 | 0.204 | -0.133 | 0.643 | 0.191 | 0.600 | 0.351 | -0.363 | 0.304 | 0.531 | 0.406 | 0.478 | 1.000 | -0.140 | -0.154 | -0.187 | 0.279 | -0.011 | 0.074 | -0.308 |
| **Dra** | -0.449 | -0.289 | -0.481 | 0.577 | 0.392 | -0.378 | -0.690 | -0.560 | -0.024 | 0.733 | -0.577 | -0.618 | -0.289 | -0.201 | -0.140 | 1.000 | 0.603 | -0.264 | -0.381 | 0.930 | 0.191 | 0.044 |
| **Urb** | -0.384 | -0.371 | -0.523 | 0.355 | 0.334 | -0.330 | -0.258 | -0.463 | 0.271 | 0.710 | -0.838 | -0.307 | -0.371 | -0.227 | -0.154 | 0.603 | 1.000 | -0.625 | -0.713 | 0.506 | 0.179 | 0.149 |
| **Imper** | -0.024 | 0.341 | 0.114 | -0.131 | -0.175 | -0.124 | -0.027 | -0.009 | -0.269 | -0.162 | 0.521 | 0.083 | 0.341 | 0.225 | -0.187 | -0.264 | -0.625 | 1.000 | 0.600 | -0.197 | 0.027 | -0.266 |
| **Foss** | 0.298 | 0.565 | 0.562 | 0.021 | 0.174 | 0.309 | 0.314 | 0.543 | -0.163 | -0.479 | 0.872 | 0.139 | 0.565 | 0.380 | 0.279 | -0.381 | -0.713 | 0.600 | 1.000 | -0.232 | 0.132 | -0.385 |
| **Cep** | -0.387 | -0.184 | -0.341 | 0.589 | 0.538 | -0.258 | -0.634 | -0.403 | -0.048 | 0.624 | -0.469 | -0.593 | -0.184 | -0.132 | -0.011 | 0.930 | 0.506 | -0.197 | -0.232 | 1.000 | 0.340 | -0.066 |
| **Sewa** | -0.142 | 0.387 | -0.160 | 0.211 | 0.482 | -0.125 | -0.112 | 0.059 | -0.167 | 0.258 | 0.033 | -0.424 | 0.387 | 0.311 | 0.074 | 0.191 | 0.179 | 0.027 | 0.132 | 0.340 | 1.000 | -0.082 |
| **Flow** | -0.014 | 0.066 | -0.083 | 0.080 | -0.177 | 0.068 | -0.010 | -0.135 | -0.061 | 0.259 | -0.285 | -0.136 | 0.066 | 0.157 | -0.308 | 0.044 | 0.149 | -0.266 | -0.385 | -0.066 | -0.082 | 1.000 |

**Table S13**. R^2^ values for the structural model.

| Predictor | R^2^ for early period | R^2^ for later period |
| --- | --- | --- |
| NAPI | 0.860 | 0.771 |
| Industrial practices | 0.537 | 0.432 |
| Agricultural practices | 0.487 | 0.623 |
| P load | 0.244 | 0.254 |
| Legacy risk | 0.712 | 0.930 |
| Vegetation | / | 0.140 |
| Wetland | 0.180 | / |
| Soil organics | 0.555 | 0.366 |

**Table S14**. Significance levels for the structural model.

| Path | P values for early period | P values for later period |
| --- | --- | --- |
| NAPI → Riverine P pollution | 0.037 | 0.025 |
| NAPI → legacy risk | 0.001 | 0.001 |
| Development pattern → NAPI | 0.001 | 0.001 |
| Development pattern → Industrial practices | 0.001 | 0.020 |
| Development pattern → Agricultural practices | 0.001 | 0.001 |
| Agricultural practices → legacy risk | 0.350 | 0.016 |
| legacy risk → P pollution | 0.036 | 0.026 |
| Industrial practices → legacy risk | 0.032 | 0.251 |
| Soil organics → legacy risk | 0.517 | 0.538 |
| Meteorology → NAPI | 0.310 | 0.043 |
| Meteorology → Soil organics | 0.001 | 0.013 |
| Meteorology → Vegetation | / | 0.465 |
| Vegetation → P pollution | / | 0.550 |
| Vegetation → legacy risk | / | 0.043 |
| Vegetation → Soil organics | / | 0.045 |
| Meteorology → Wetland | 0.510 | / |
| Wetland → P pollution | 0.140 | / |
| Wetland → legacy risk | 0.038 | / |
| Wetland → Soil organics | 0.048 | / |

**Table S15.** Scenarios for the Random Forest model.

| Scenarios | Descriptions | Scenario assumptions |
| --- | --- | --- |
| BAU | Business-as-usual | Constant basin P management practices (e.g., BAU changes in P use efficiency, and woodland and wetland cover); constant fraction of the population connected to human waste productions and constant dietary structure. |
| DSI | Dietary structure improvement | Here, we considered a shift away from animal-based diets that reduces the gap between current and recommended consumption (40-75 g/capita/day; Chinese Dietary Guidelines) by 15-18% in 2050^24^, with a linear progression towards that target starting in 2020; meanwhile, we believed that increasing the crop-based food consumption can ensure total calorie consumption^24^. We achieved this target through constant per capita P consumption, reduced livestock excretion (i.e., the difference between consumption and production), and increased crop yield. |
| FER | Fossil energy consumption reduction | To reach the carbon neutrality goal in China before 2060, the proportion of fossil energy (e.g., coal, oil, and gas) will decrease to less than 41% of the energy mix by 2050^25^, with a linear progression towards that target starting in 2020 when the proportion of fossil energy consumption is about 84.1%^25^. This can directly impact the emissions of atmospheric P deposition (Supplemental Section 1.2.2). |
| PUE | P use efficiency enhancement | The regional NPE of cropland reaching the target NPE (83%) by 2050 with a linear progression towards that target starting in 2020^26^. For sub-basins where the baseline NPE (for the year 2020) calculated is higher than the target NPE, the NPE remains constant. |
| COM | Combining all of the above improved measures | Simultaneous implementation of all the above improved P management strategies. |

**Reference**

**(**1) Liu, B.; Liu, C.; Zhang, G.; Zhao, Z.; Li, S.; Hu, J.; Ding, H.; Lang, Y.; Li, X. Chemical weathering under mid- to cool temperate and monsoon-controlled climate: A study on water geochemistry of the Songhuajiang River system, northeast China. *Applied Geochemistry* **2013,** *31*, 265-278.

(2) Seki, O.; Mikami, Y.; Nagao, S.; Bendle, J. A.; Nakatsuka, T.; Kim, V. I.; Shesterkin, V. P.; Makinov, A. N.; Fukushima, M.; Moossen, H. M.; Schouten, S. Lignin phenols and BIT index distributions in the Amur River and the Sea of Okhotsk: Implications for the source and transport of particulate terrestrial organic matter to the ocean. *Progress in Oceanography* **2014,** *126*, 146-154.

(3) Gao, Y.; Tian, Y.; Zhan, W.; Li, L.; Sun, H.; Zhao, T.; Zhang, H.; Meng, Y.; Li, Y.; Liu, T.; Ding, J. Characterizing legacy nitrogen-induced time lags in riverine nitrogen reduction for the Songhuajiang River Basin: Source analysis, spatio-seasonal patterns, and impacts on future water quality improvement. *Water Research* **2023,** *242*, 120292.

(4) Liu, J.; Gu, W.; Liu, Y.; Zhang, C.; Li, W.; Shao, D. Dynamic characteristics of net anthropogenic phosphorus input and legacy phosphorus reserves under high human activity - A case study in the Jianghan Plain. *Science of The Total Environment* **2022,** *836*, 155287.

(5) Han, Y.; Yu, X.; Wang, X.; Wang, Y.; Tian, J.; Xu, L.; Wang, C. Net anthropogenic phosphorus inputs (NAPI) index application in Mainland China. *Chemosphere* **2013,** *90*, 329-337.

(6) Wang, Y.; Xie, X.; Liu, C.; Wang, Y.; Li, M. Variation of net anthropogenic phosphorus inputs (NAPI) and riverine phosphorus fluxes in seven major river basins in China. *Science of The Total Environment* **2020,** *742*, 140514.

(7) Hu, M.; Liu, Y.; Zhang, Y.; Shen, H.; Yao, M.; Dahlgren, R. A.; Chen, D. Long-term (1980-2015) changes in net anthropogenic phosphorus inputs and riverine phosphorus export in the Yangtze River basin. *Water Research* **2020,** *177*, 115779.

(8) Han, Y.; Li, X.; Nan, Z. Net Anthropogenic Phosphorus Accumulation in the Beijing Metropolitan Region. *Ecosystems* **2011,** *14*, 445-457.

(9) Hu, M.; Liu, Y.; Wang, J.; Dahlgren, R. A.; Chen, D. A modification of the Regional Nutrient Management model (ReNuMa) to identify long-term changes in riverine nitrogen sources. *Journal of Hydrology* **2018,** *561*, 31-42.

(10) Chen, D.; Huang, H.; Hu, M.; Dahlgren, R. A. Influence of lag effect, soil release, and climate change on watershed anthropogenic nitrogen inputs and riverine export dynamics. *Science of The Total Environment* **2014,** *48*, 5683-5690.

(11) Li, L.; Blomberg, A. J.; Stern, R. A.; Kang, C.-M.; Papatheodorou, S.; Wei, Y.; Liu, M.; Peralta, A. A.; Vieira, C. L. Z.; Koutrakis, P., Predicting Monthly Community-Level Domestic Radon Concentrations in the Greater Boston Area with an Ensemble Learning Model. *Environmental Science & Technology* **2021,** *55*, 7157-7166.

(12) Hair, J. F.; Ringle, C. M.; Sarstedt, M. PLS-SEM: Indeed a Silver Bullet. *Journal of Marketing Theory and Practice* **2014,** *19*, 139-152.

(13) Liang, J.; Tang, W.; Zhu, Z.; Li, S.; Wang, K.; Gao, X.; Li, X.; Tang, N.; Lu, L.; Li, X. Spatiotemporal variability and controlling factors of indirect N_2_O emission in a typical complex watershed. *Water Research* **2023,** *229*, 119515.

(14) Chen, D.; Shen, H.; Hu, M.; Wang, J.; Zhang, Y.; Dahlgren, R. A. Legacy Nutrient Dynamics at the Watershed Scale: Principles, Modeling, and Implications. *Advances in Agronomy* **2018,** *149*, 237-313.

(15) Boardman, E.; Danesh-Yazdi, M.; Foufoula-Georgiou, E.; Dolph, C. L.; Finlay, J. C. Fertilizer, landscape features and climate regulate phosphorus retention and river export in diverse Midwestern watersheds. *Biogeochemistry* **2019,** *146*, 293-309.

(16) Van Meter, K. J.; McLeod, M. M.; Liu, J.; Tenkouano, G. T.; Hall, R. I.; Van Cappellen, P.; Basu, N. B. Beyond the Mass Balance: Watershed Phosphorus Legacies and the Evolution of the Current Water Quality Policy Challenge. *Water Resources Research* **2021,** *57*, e2020WR029316.

(17) Sanford, W. E.; Pope, J. P. Quantifying groundwater's role in delaying improvements to Chesapeake Bay water quality. *Environmental Science & Technology* **2013,** *47*, 13330-13338.

(18) Tesoriero, A. J.; Duff, J. H.; Saad, D. A.; Spahr, N. E.; Wolock, D. M. Vulnerability of streams to legacy nitrate sources. *Environmental Science & Technology* **2013,** *47*, 3623-3629.

(19) Liu, J.; Van Meter, K. J.; McLeod, M. M.; Basu, N. B. Checkered landscapes: hydrologic and biogeochemical nitrogen legacies along the river continuum. *Environmental Research Letters* **2021,** *16*, 115006.

(20) Castellano, M. J.; David, M. B. Long-term fate of nitrate fertilizer in agricultural soils is not necessarily related to nitrate leaching from agricultural soils. *Proceedings of the National Academy of Sciences of the USA* **2014,** *111*, E766.

(21) Merrill, N. H.; Piscopo, A. N.; Balogh, S.; Furey, R. P.; Mulvaney, K. K. When, where, and how to intervene? Trade-offs between time and costs in coastal nutrient management. *Journal of the American Water Resources Association* **2021,** *57*, 328-343.

(22) Xi, Y.; Peng, S.; Liu, G.; Ducharne, A.; Ciais, P.; Prigent, C.; Li, X.; Tang, X. Trade-off between tree planting and wetland conservation in China. *Nature Communications* **2022,** *13*, 1967.

(23) Hong, B.; Swaney, D. P.; Howarth, R. W. Estimating net anthropogenic nitrogen inputs to U.S. watersheds: comparison of methodologies. *Environmental Science & Technology* **2013,** *47*, 5199-5207.

(24) Ren, M.; Huang, C.; Wu, Y.; Deppermann, A.; Frank, S.; Havlík, P.; Zhu, Y.; Fang, C.; Ma, X.; Liu, Y.; Zhao, H.; Chang, J.; Ma, L.; Bai, Z.; Xu, S.; Dai, H. Enhanced food system efficiency is the key to China’s 2060 carbon neutrality target. *Nature Food* **2023,** *4*, 552-564.

(25) Dai, H.; Su, Y.; Kuang, L.; Liu, J.; Gu, D.; Zou, C. Contemplation on China’s Energy-Development Strategies and Initiatives in the Context of Its Carbon Neutrality Goal. *Engineering* **2021,** *7*, 1684-1687.

(26) Zou, T.; Zhang, X.; Davidson, E. A. Global trends of cropland phosphorus use and sustainability challenges. *Nature* **2022,** *611*, 81-87.

(27) Kusmer, A. S.; Goyette, J. O.; MacDonald, G. K.; Bennett, E. M.; Maranger, R.; Withers, P. J. A. Watershed buffering of legacy phosphorus pressure at a regional scale: a comparison across space and time. *Ecosystems* **2019***, 22*, 91-109.
